# GABP Promotes Mesangial Cell Proliferation and Renal Fibrosis Through GLI1 in Diabetic Nephropathy

**DOI:** 10.1002/advs.202407462

**Published:** 2025-02-22

**Authors:** Lei Du, Sijie Liu, Yinfei Lu, Dongxue Ren, Xiujuan Yu, Yue Hu, Tingting Yang, Qun Yang, Jingxian Ming, Jiawei Zhang, Xiaoxing Yin, Qian Lu

**Affiliations:** ^1^ Jiangsu Key Laboratory of New Drug Research and Clinical Pharmacy Xuzhou Medical University China

**Keywords:** diabetic nephropathy, GABP, GLI1, mesangial cell proliferation, renal fibrosis

## Abstract

Abnormal proliferation of mesangial cells is a hallmark of diabetic nephropathy (DN). However, the cellular signaling mechanisms that regulate this proliferation remain poorly understood. In this study, it is demonstrated that GA‐binding protein (GABP), a member of the ETS family of transcription factors composed of GABPα and GABPβ, plays a significant role in the development of renal fibrosis by modulating mesangial cell proliferation. Notably, the deficiency of GABP in mesangial cells inhibits hyperglycemia‐induced proliferation and mitigates renal fibrosis in a murine model of type 2 diabetes mellitus (T2DM). RNA sequencing analysis identifies GLI Family Zinc Finger 1 (GLI1) as the principal downstream effector of GABP in diabetic mice, serving as a crucial regulator of the G1/S transition within the cell cycle. Subsequent investigations have demonstrated that GABP interacts with the GLI1 promoter, facilitating mesangial cell proliferation via GLI1‐dependent pathways. This is evidenced by the fact that GLI1 knockdown abrogates the proliferation of mesangial cells with GABP overexpression. Consequently, GABP emerges as a pivotal regulator of renal fibrosis and represents a promising therapeutic target for the treatment of diabetic nephropathy.

## Graphical abstract

GABP is found to promote mesangial cell proliferation and ECM deposition through GLI1, while inhibiting GABP or GLI1 prevents the progression of DN by reducing ECM and fibrosis. The research results indicate that a potential therapeutic approach based on inhibiting mesangial cell proliferation and ECM accumulation can effectively treat DN, while GABP can serve as an early predictor of DN disease occurrence.

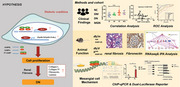

## 1 Introduction

DN, a significant chronic complication of diabetes, affects approximately one‐third of patients with T2DM.^[^ ^1^ ^]^ DN is the primary cause of end‐stage renal disease in adults and is often characterized by gradual onset and rapid progression.^[^ ^2^ ^]^ Patients typically exhibit proteinuria or decreased renal function upon diagnosis, indicating irreversible renal damage.^[^ ^3^ ^]^ However, the absence of reliable early diagnostic biomarkers in clinical practice remains a significant obstacle to the effective treatment of DN.^[^ ^4^ ^]^

Previous research has established that the initial pathological alterations in DN encompass mesangial cell proliferation and extracellular matrix (ECM).^[^ ^5^, ^6^ ^]^ Activated mesangial cells produce excessive extracellular matrix components such as type I, III, and IV collagen, fibronectin, and inflammatory mediators. This results in the expansion of the mesangial matrix, basement membrane thickening, glomerular sclerosis, and renal interstitial fibrosis.^[^ ^7^ ^]^ Renal fibrosis reduces glomerular filtration and eventually culminates in irreversible renal failure.^[^ ^8^ ^]^ Transcription factors regulate the progression of renal fibrosis.^[^ ^9^, ^10^, ^11^ ^]^ However, the precise mechanisms and their significance in the early diagnosis of DN remain unclear.

We conducted a proteomic analysis of the kidneys of DN mice with renal fibrosis and non‐diabetic control mice. We identified a highly upregulated GABP for DNA‐binding transcription factor activity in the GO terms. GABP is an Ets transcription factor that activates transcription. GABP is a heterotetramer composed of GABPα and GABPβ subunits necessary to generate a functional complex that binds DNA and activates gene transcription.^[^ ^12^ ^]^ GABP regulates lineage‐restricted genes, ribosomal and mitochondrial genes, regulatory genes, synapse‐specific genes, and genes that control the cellular growth and differentiation of various tissues.^[^ ^13^, ^14^, ^15^, ^16^ ^]^ GABP is widely expressed and plays pivotal roles in various crucial physiological functions, pathophysiological states, and fundamental cellular activities. The transcriptional regulatory function of GABP regulates important processes such as mitochondrial function, protein synthesis, and cell cycle events.^[^ ^17^, ^18^, ^19^ ^]^ GABP, as a transcription factor, regulates the expression of these effector genes to affect the phenotype of diseases such as cancer and diabetes. The absence of GABPβ in B lymphocytes affects their growth and death, highlighting the role of GABP cell proliferation. GABPα knockdown causes podocyte apoptosis, and GABPβ1 knockdown inhibits renal cancer cell proliferation, indicating the involvement of GABP in regulating renal cell growth, proliferation, and apoptosis. However, the expression and function of GABP in DN renal fibrosis remain unknown.

Therefore, we further investigated the role and molecular mechanisms of GABP in renal fibrosis and mesangial cell proliferation using biological methods, such as transcriptome analysis. We observed that GABP directly regulates the transcription of GLI, exacerbating the proliferation of glomerular mesangial cells (GMCs) and the accumulation of ECM, leading to renal fibrosis. In addition, a retrospective clinical analysis observed that GABP may serve as an early diagnostic marker for DN, thereby enhancing the performance of DN prediction models. To the best of our knowledge, this is the first study to elucidate the mechanism and clinical significance of the action of GABP on mesangial cell proliferation and DN renal fibrosis. Our study provides a new theoretical basis for the mechanism of DN renal fibrosis and new ideas for the prevention and early diagnosis of DN.

## 2 Results

### 2.1 Identification of GABP as a Potential Transcriptional Regulator of Diabetic Nephropathy From Proteomic Analysis

**Figure 1:**
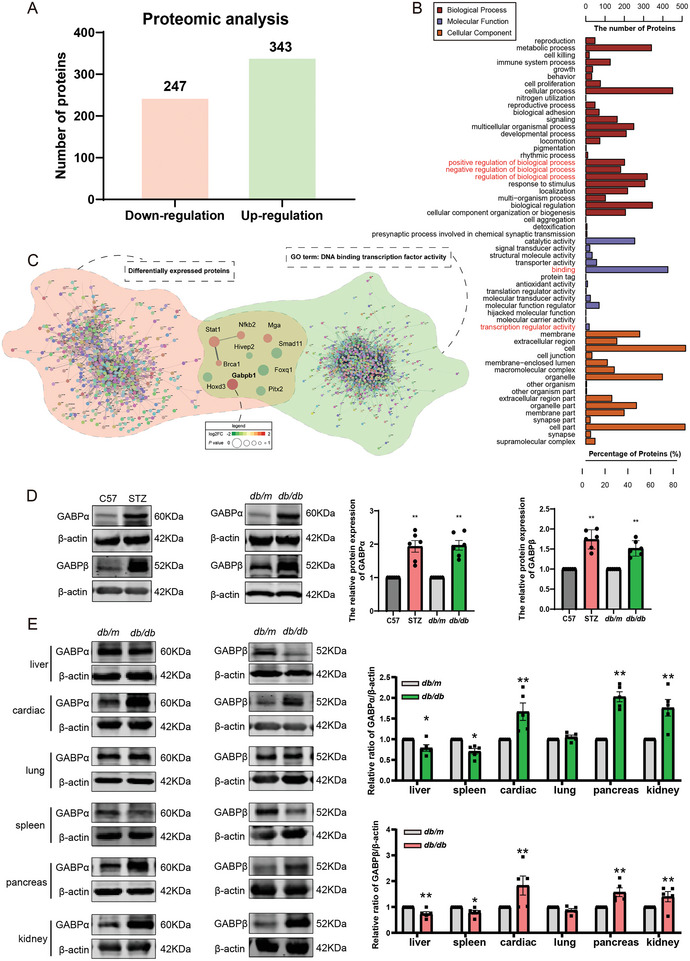
Identification of GABP as a Potential Transcriptional Regulator of Diabetic Nephropathy from Proteomic Analysis. A) Proteomic analysis of the differentially expressed proteins in the kidneys of STZ‐induced diabetic mice; B) Gene Ontology analysis of cellular component, biological processes, and molecular function of differently expressed proteins; C) Venn diagram of Protein‐Protein interaction network between differentially expressed proteins and proteins possessing DNA binding transcription factor activity, as defined by the GO term of Mouse Genome Informatics (MGI); D) Expression of GABPα and GABPβ in kidney of two different diabetic mouse models, *n* = 6; E) Expression level of GABP in major organs of *db/m* and *db/db* mice, *n* = 5. Data are expressed as mean  ±  s.e.m. Statistical significance was assessed using a two‐way ANOVA with Tukey's test, ^*^ *p* < 0.05, ^**^ *p* < 0.01, compared to C57 or *db/m*.

To identify the proteins that were differentially expressed in DN, we performed Tandem Mass Tag (TMT) quantitative proteomics to identify differently expressed proteins (DEPs) in kidney tissues from C57 (non‐diabetic control mice) and STZ (DN model mice) groups. The STZ mouse model and C57 controls are verified in Figure S1A–F (Supporting Information). Comparing the C57 and STZ groups, after normalizing and filtering all data (Fold Change > 1.2 or < 0.83, *P* value < 0.05), 343 proteins were upregulated, and 247 proteins were down‐regulated in the kidney tissue (**Figure** **1**A). GO analysis showed that the binding term enriched most DEPs in the molecular function category (Figure 1B). Because transcriptional regulation plays an important role in cell proliferation, we selected proteins enriched under the activity of DNA‐binding transcription factors in the Mouse Genome Informatics (MGI) database for further analysis. The results showed that GABP was the protein with the largest fold change at the intersection of DEPs and DNA‐binding transcription factors (Figure 1C). In two types of DN model mice, including those induced by a high‐fat diet with STZ and transgenic *db/db* mice, GABP protein levels in the renal cortex were significantly higher compared to controls, aligning with proteomic findings. This suggests a general increase in GABP protein expression in these DN models (Figure 1D). Furthermore, we measured GABPα and GABPβ levels in the heart, liver, spleen, lungs, pancreas, and kidneys of *db/m* and *db/db* mice using western blotting. The results showed that GABP was expressed in all these tissues. However, the expression of GABP in the heart, pancreas, and kidneys of *db/db* mice was significantly higher than that in control *db/m* mice, and the expression pattern of GABP in the kidneys of *db/db* mice was the same as that in STZ mice (Figure 1E).

### 2.2 GABP Expression is Specifically Augmented in Mesangial Cells Under Diabetic Conditions

**Figure 2:**
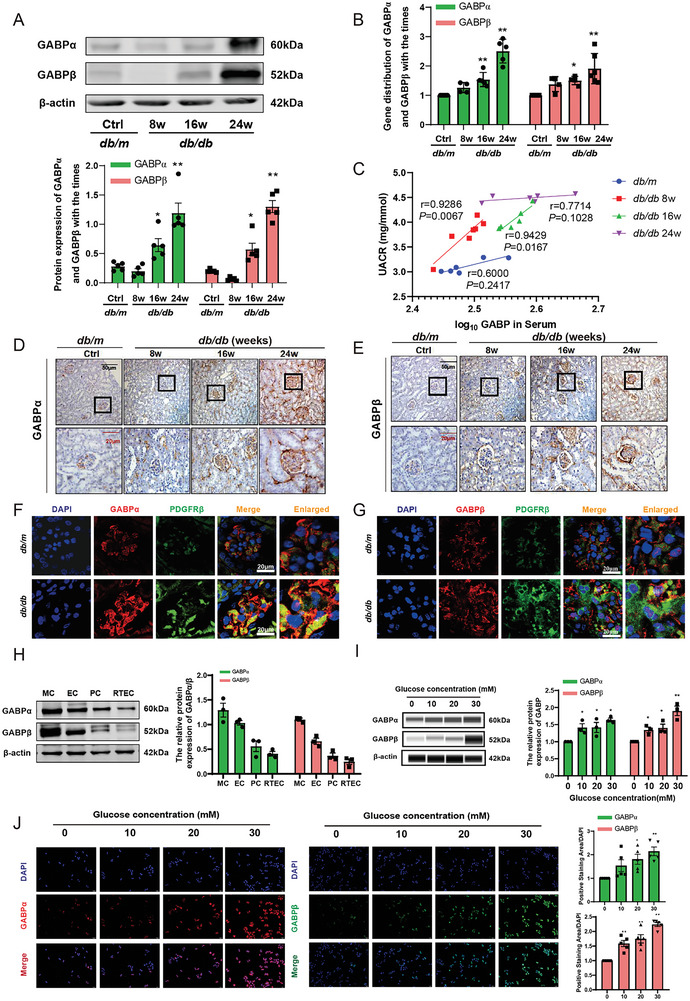
GABP Expression is Specifically Augmented in Mesangial Cells under Diabetic Conditions. A) The protein and B) mRNA expression levels of GABPα and GABPβ in *db/m* and *db/db* mice at 8, 16, and 24 weeks of age, *n* = 5. C) Correlation analysis between GABP expression in serum and UACR in *db/m* and *db/db* mice at 8, 16, and 24 weeks of age, *n* = 6. D,E) Immunohistochemical analysis of GABPα and GABPβ expression in the kidney of *db/m* and *db/db* mice at 8, 16, and 24 weeks of age (Scale bar: 50 and 20 µm), *n* = 6. F,G) Colocalization of GABPα/GABPβ and PDGFRβ by immunofluorescence in glomerular mesangial cells (PDGFRβ, green fluorescence; GABPα/GABPβ, red fluorescence; Scale bar: 20 µm). H) The protein expression levels of GABP in GMCs, endothelial cells (EC), podocytes (PC), and renal tubular epithelial cells (RTEC), *n* = 3. I) The protein expression levels of GABP in glomerular mesangial cells cultured with different glucose concentrations, *n* = 3. J) Immunofluorescence was used to detect the protein expression of GABP in glomerular mesangial cells cultured with 0, 10, 20, and 30 mm glucose concentrations (GABPβ, green fluorescence; GABPα, red fluorescence; Scale bar: 100 µm), *n* = 5. Data are expressed as mean  ±  s.e.m. Statistical significance was assessed using a one‐way ANOVA with Tukey's test, ^*^ *p* < 0.05, ^**^ *p* < 0.01, compared to *db/m* or 0 mM glucose concentration.

We observed that GABP was upregulated in the kidneys of *db/db* mice of different ages, which was further confirmed using western blotting (**Figure** **2**A), qPCR (Figure 2B), and immunohistochemical staining (Figure 2D,E). Correlation analysis showed that the expression of GABP in the serum increased with age and was significantly positively correlated with urinary albumin/creatinine ratio (UACR) in mice. In the early stages of diabetes in *db/db* mice, such as at 8 or 16 weeks of age, GABP may be an early marker of diabetic renal function damage (Figure 2C). Immunofluorescence analysis of *db/db* mice revealed the colocalization of GABP with the mesangial cell marker PDGFRβ. Importantly, an increased expression of GABP was primarily observed in the mesangial regions of the glomeruli in *db/db* mice (Figure 2F,G). We identified the expression of GABP in renal parenchymal cells, specifically in GMCs, endothelial cells (ECs), podocytes (PCs), and renal tubular epithelial cells (RTECs). Notably, GABP expression was found to be significantly more abundant in mesangial cells (Figure 2H). Subsequently, mesangial cells were treated with 0, 10, 20, and 30  mM glucose, and western blot analysis revealed that the expression of GABP in GMCs gradually increased (Figure 2I). Immunofluorescence results showed that the expression of GABP increased with glucose concentration and was mainly concentrated in the nucleus, consistent with the functional location of the GABP transcription factors (Figure 2J).

### 2.3 GABP Overexpression Promotes Mesangial Cell Proliferation and Aggravates Renal Fibrosis in *db/m* Mice

**Figure 3:**
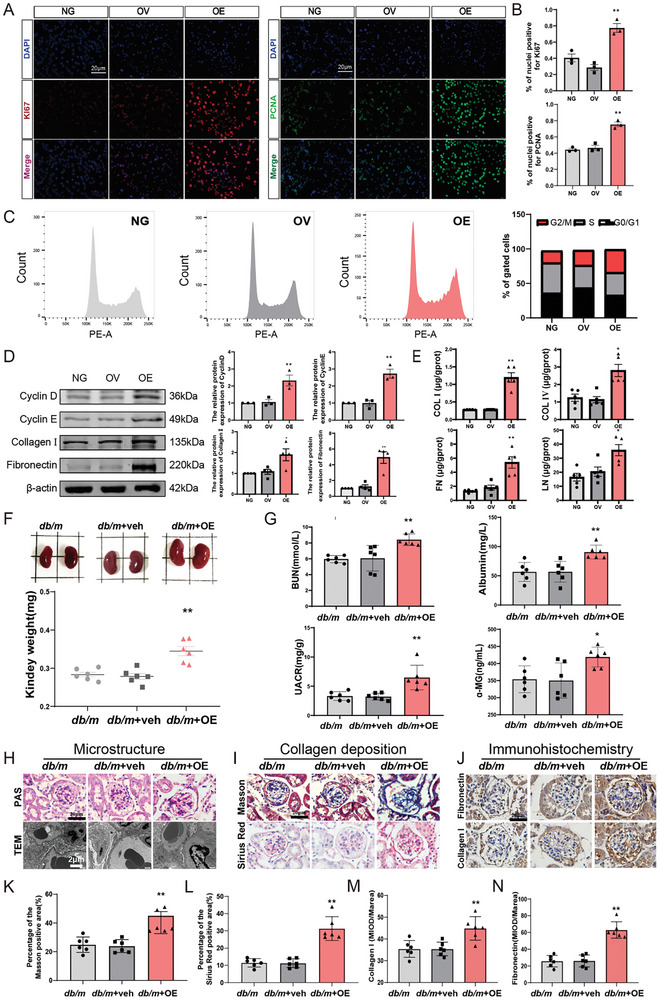
GABP Overexpression Promotes Mesangial Cell Proliferation and Aggravates Renal Fibrosis of *db/m* Mice. A) Immunofluorescence and B) its quantitative analysis was used to detect the expression of PCNA, Ki67 in the mesangial cells (PCNA, green fluorescence; Ki67, red fluorescence; Scale bar: 20 µm); *n* = 3; C) Cell cycle of mesangial cells by flow cytometry. *n* = 3; D) The protein expression level of Cyclin D, Cyclin E, FN, and COL I in mesangial cells by western blot, *n* = 3; E) The expressions of LN, FN, COL I and COL IV in mesangial cells by ELISA, *n* = 5; F) Kidney volume and weight of mice, *n* = 6; G) BUN, albumin, UACR and α‐MG of mice, *n* = 6; H) PAS staining (Positive area: red; Scale bar: 20 µm) and transmission electron microscopy (Scale bar: 2 µm) of kidney tissue in mice; I) Sirius red staining (Positive area: red) and Masson staining (Positive area: blue) of kidney tissue in mice, (Scale bar: 20 µm); J) The expression of FN and Collagen I in glomeruli by immunohistochemical staining (Positive area: brown; Scale bar: 20 µm); K,L) The quantitation of Masson and Sirius red postive staining areas, *n* = 6; M,N) The quantification of Fibronectin and Collagen I expression, *n* = 6. NG: normal mesangial cell; OV: normal mesangial cell with vector; OE: normal mesangial cell with GABPα/β overexpression lentivirus; *db/m*: normal control mice; *db/m*+Veh: *db/m* mice with vector; *db/m*+OE: *db/m* mice with intra‐renal injection of GABPα/β overexpression adeno‐associated virus. Data are expressed as mean  ±  s.e.m. Statistical significance was assessed using a one‐way ANOVA with Tukey's test, ^*^ *p* < 0.05, ^**^ *p* < 0.01, compared to OV or *db/m + V*eh.

Mesangial cell proliferation is an important factor in DN pathogenesis. To further clarify the influence of GABP on the proliferation of GMCs, GABPα/β overexpression (OE) models of GMCs were generated by lentivirus infection (Figure S2A–H, Supporting Information). Immunofluorescence results showed that the positive expression of PCNA and Ki67 in GMCs increased significantly with GABPα/β overexpression (**Figure** **3**A,B). We analyzed the cell cycle distribution. GABPα/β OE increased the percentage of mesangial cells in the G2/M phase (Figure 3C). Western blot analysis was performed to quantify cyclin D1 and Cyclin E levels, which are effector proteins responsible for GMCs proliferation.^[^ ^20^ ^]^ The results showed that compared to the normal mesangial cell with vector (OV) group, the protein expression of Cyclin D1, Cyclin E, Fibronectin (FN), and Collagen I (COL I) increased in the OE group (Figure 3D). An important effect of mesangial cells proliferation is the promotion of ECM accumulation. To explore the role of GABP in ECM generation in GMCs, the secretion of laminin (LN), FN, COL I, and Collagen IV (COL IV) into the extracellular fluid was detected using ELISA (Figure 3E). Compared with the OV group, the secretion of FN, LN, COL I, and COL IV in the OE group increased and accumulated in the intercellular space. These results indicate that GABP promotes the secretion and accumulation of ECM components in GMCs.

To investigate the role of GABP in renal fibrosis, we delivered adeno‐associated virus (AAV) into mouse kidneys via renal parenchymal injection. The expression of AAV‐green fluorescent protein (GFP) in the kidney was confirmed 2 weeks after injection and remained stable after 4 weeks (Figure S3A–E, Supporting Information). The kidney‐specific knockdown or overexpression of GABP does not significantly affect body weight or fasting blood glucose levels (FBG) (Figure S3F, Supporting Information). Furthermore, the observed increase in kidney weight, blood urea nitrogen (BUN) levels, urinary albumin, urinary albumin‐to‐creatinine ratio (UACR), and urinary α‐Microglobulin (α‐MG) (Figure 3F,G) in mice suggests that the overexpression of GABP may induce renal dysfunction, primarily through glomerular damage, resulting in the development of albuminuria. Periodic acid‐Schiff (PAS) staining and transmission electron microscopy (TEM) analyses revealed that in comparison to the *db/m*+veh group (*db/m* mice with vector), the *db/m*+OE (*db/m* mice with GABPα/β overexpression AAV) group exhibited enlarged glomeruli, a thickened basement membrane of the glomerular capillary loop, and proliferation of mesangial cells and matrix (Figure 3H). Concurrently, for Sirius Red and Masson's trichrome staining, there was an observed increase in the positive area within the glomeruli of mice in *db/m*+OE group (Figure 3I–L). Immunohistochemical (IHC) analysis revealed an elevated accumulation of FN and COL I in the glomeruli of mice in *db/m*+OE group (Figure 3J–N). Collectively, these findings indicate that the overexpression of GABP facilitates the progression of diabetic kidney disease.

### 2.4 GABP Knockdown Inhibits Mesangial Cell Proliferation Under High Glucose Conditions and Ameliorates Renal Fibrosis in *db/db* Mice

**Figure 4:**
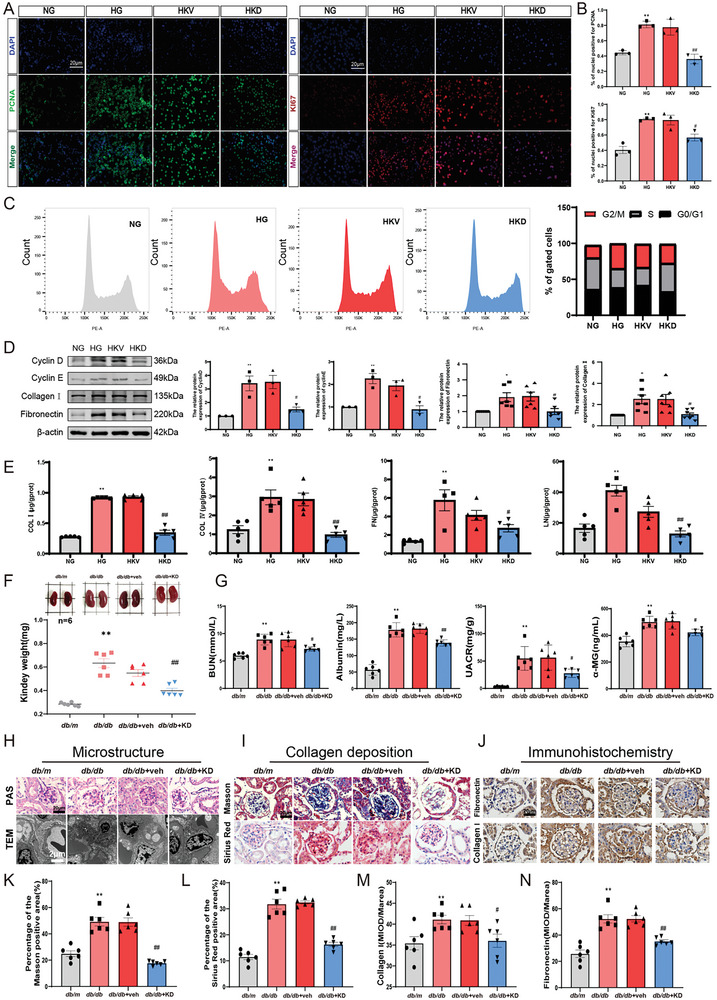
GABP Knockdown Inhibits Mesangial Cell Proliferation under High Glucose Conditions and Ameliorates Renal Fibrosis of *db/db* Mice. A) Immunofluorescence and B) its quantitative analysis was used to detect the expression of PCNA, Ki67 in the mesangial cells (PCNA, green fluorescence; Ki67, red fluorescence; Scale bar: 20 µm), *n* = 3. C) Cell cycle of mesangial cells by flow cytometry. *n* = 3. D) The protein expression levels of Cyclin D, Cyclin E, FN, and COL I in mesangial cells by western blot, *n* = 3. E) The expressions of LN, FN, COL I, and COL IV by ELISA, *n* = 5. F) Kidney volume and weight of mice, *n* = 6. G) BUN, albumin, UACR, and α‐MG of mice, *n* = 6. H) PAS staining (Positive area: red Scale bar: 20 µm) and transmission electron microscopy (Scale bar: 2 µm) of kidney tissue in mice. I) Sirius red staining (Positive area: red) and Masson staining (Positive area: blue) of kidney tissue in mice, (Scale bar: 20 µm). J) The expression of FN and Collagen I in glomeruli by immunohistochemical staining (Positive area: brown; Scale bar: 20 µm). K,L) The quantitation of Masson and Sirius red postive staining areas, *n* = 6. M,N) The quantification of Fibronectin and Collagen I expression, *n* = 6. NG: normal mesangial cell; HG: mesangial cell with 30 mM glucose; HKV: high glucose cultured mesangial cell with vector; HKD: high glucose cultured mesangial cell with GABPβ‐knockdown lentivirus; *db/m*: normal control mice; *db/db*: diabetic model mice; *db/db*+veh: *db/db* mice with intra‐renal injection of vector; *db/db*+KD: *db/db* mice with intra‐renal injection of GABPβ‐knockdown adeno‐associated virus. Data are expressed as mean  ±  s.e.m. Statistical significance was assessed using a one‐way ANOVA with Tukey's test, ^*^ *p* < 0.05, ^**^ *p* < 0.01, compared to NG or *db/m*, ^#^ *p* < 0.05, ^##^ *p* < 0.01, compared to HKV or *db/db* + veh.

Subsequent to the knockdown of GABP in mesangial cells cultured under high glucose conditions (Figure S2A–H, Supporting Information), the expression of cell proliferation nuclear antigens Ki67 and PCNA was assessed using immunofluorescence assays (**Figure** **4**A,B). Additionally, cell cycle analysis was conducted via flow cytometry (Figure 4C), while the expression of cell cycle‐related and ECM‐related proteins was evaluated (Figure 4D). In comparison to the control group, there was a significant increase in the positive area of PCNA and Ki67, as well as a marked elevation in the protein expression levels of Cyclin D and Cyclin E. Additionally, the proportion of mesangial cells in the G2/M phase was elevated, and there was an accumulation of LN, FN, COL I, and COL IV (Figure 4E). These findings suggest that high glucose conditions can induce mesangial cell proliferation, resulting in the accumulation of the extracellular matrix. Notably, the knockdown of GABP significantly inhibited the proliferation of mesangial cells and the accumulation of the extracellular matrix induced by high glucose.

Further in vivo experiments showed that *db/db* mice had significantly higher kidney weight, BUN, albumin, UACR, and α‐MG levels compared to the *db/m* group (Figure 4F,G). These results suggest a marked decline in renal function and the development of proteinuria in diabetic mice. PAS and TEM showed basement membrane thickening in *db/db* mice (Figure 4H). Additionally, there was an increase in the areas positive for Sirius Red and Masson's trichrome staining in *db/db* mice (Figure 4I‐L). Immunohistochemistry revealed elevated FN and collagen I protein levels in the glomeruli of *db/db* mice, indicating renal fibrosis and ECM protein deposition (Figure 4J–N). GABP‐knockdown‐*db/db* mice exhibited significantly reduced levels of BUN, Albumin, UACR, urinary α‐MG, and kidney fibrosis markers compared to the *db/db*+veh group. They also showed lower protein levels of fibrosis‐related molecules. This indicates that the suppression of GABP expression may decelerate renal fibrosis and extracellular matrix accumulation associated with diabetic nephropathy, thereby enhancing renal function in murine models.

### 2.5 RNA‐Seq Identified GLI1 as the Target Gene for GABP

**Figure 5:**
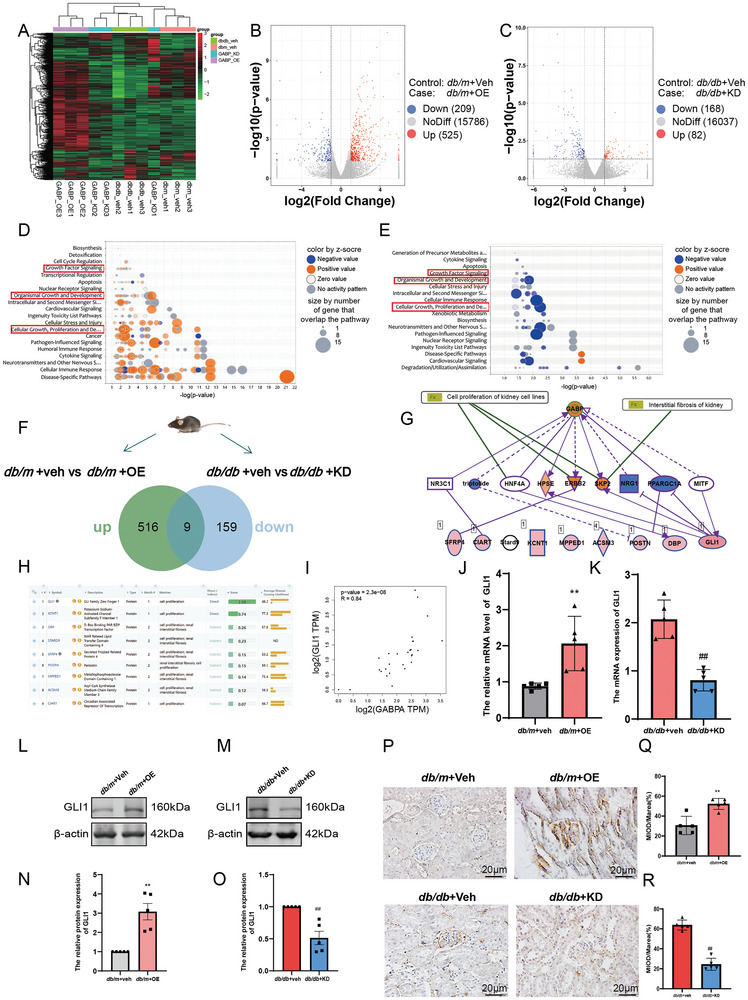
RNA‐seq Identified GLI1 as the Target Gene for GABP. A) Cluster heat map analysis for transcriptomic analysis; B,C) Volcanic map for transcriptomic analysis; D) Classical pathway analysis of GABP overexpress; E) Classical pathway analysis of GABP knockdown; F) Differential gene Venn diagram; G) Differential gene IPA network diagram; H) The nine differential genes generate Gene‐phenotype connections are mapped by VarElect; I) Correlation analysis between GABPα and GLI1; J,K) The mRNA expression levels of GLI1 in the kidney of mice, *n* = 5; L,M) The protein expression level of GLI1 in the kidney of mice by western blot, *n* = 5; N,O) Quantitative analysis of Figure L and M, *n* = 5; P) Immunohistochemical staining (Positive area: brown; Scale bar: 20 µm) and Q,R) its quantitative analysis of GLI1 in the kidney of mice, *n* = 5. *db/m*: normal control mice; *db/db*: diabetic model mice; *db/db*+veh: *db/db* mice with intra‐renal injection of vector; *db/db*+KD: *db/db* mice with intra‐renal injection of GABPβ‐knockdown adeno‐associated virus. Data are expressed as mean  ±  s.e.m. Statistical significance was assessed using an unpaired t‐test. ^*^ *p* < 0.05, ^**^ *p* < 0.01, compared to *db/m+*veh, ^#^ *p* < 0.05, ^##^ *p* < 0.01, compared to *db/db*+veh.

We performed RNA‐seq‐based transcriptomic analysis to evaluate the differential gene expression in the kidneys of *db/m*+OE and *db/db*+KD mice. The cluster analysis heat map showed the genetic changes (**Figure** **5**A). Compared to *db/m*+veh mice, there were 734 differentially expressed genes, 525 upregulated genes, and 209 downregulated genes (Figure 5B). Compared to *db/db*+veh mice, 82 genes were upregulated, and 168 genes were downregulated (Figure 5C). In addition, the classic pathway map in the IPA database showed that overexpression of GABP‐activated pathways such as cell growth, proliferation, and organ development, while knockdown of GABP inhibited these pathways (Figure 5D‐E, red indicates active, blue indicates inhibited). Among the differentially expressed genes, nine differentially expressed genes were upregulated by GABP overexpression and downregulated by GABP knockdown (Figure 5F; Table S1, Supporting Information). IPA database analysis showed that GABP interacts with nine proteins, including GLI1, DBP, and SFRP4, in cell proliferation and renal interstitial fibrosis‐related pathways (Figure 5G). In addition, we used the VarElect database to search for disease/phenotype‐dependent gene variant prioritization of nine related genes; GLI1 scores related to proliferation and development ranked first (Figure 5H). The GEPIA database shows that the correlation coefficient between GLI1 and GABPα was 0.84, ranked first (Figure 5I; Figure S4A–H, Supporting Information). GLI1 expression was upregulated after GABP overexpression and downregulated after GABP knockdown (Figure 5J–O). In addition, in the kidneys of mice overexpressing or knocking down GABP, the expression of GLI1 was mainly concentrated in the glomerular region, as confirmed using IHC (Figure 5P‐R). Therefore, GLI1 was selected as the downstream target gene of GABP for further experiments.

### 2.6 GABP Positively Associates with GLI1 and Directly Binds to its Promoter to Promote Transcription

**Figure 6:**
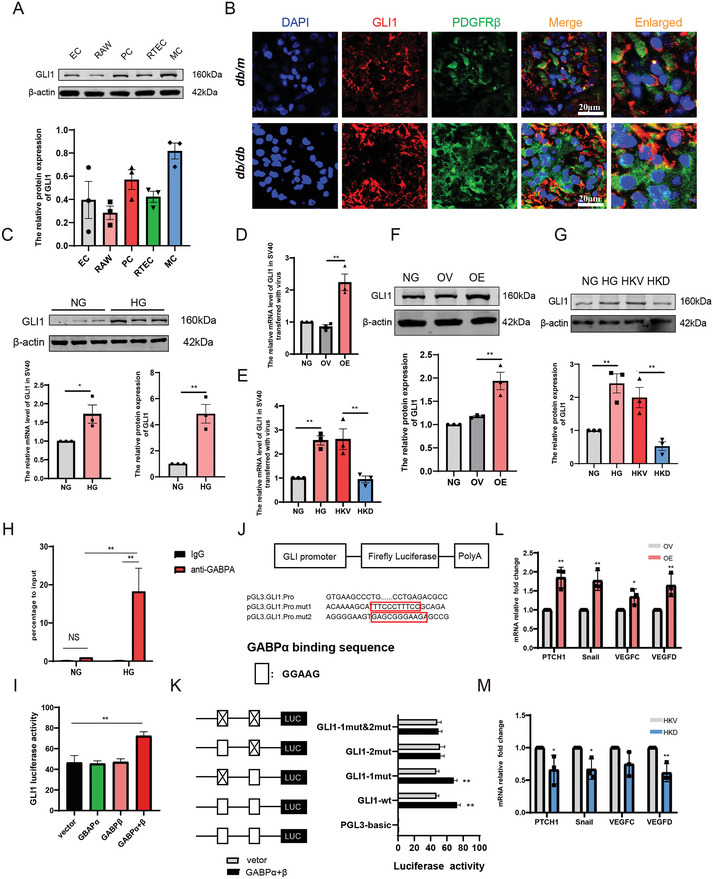
Specific Expression of GLI1 in Mesangial Cells and Positive Regulation of GLI1 Expression by GABP in vivo. A) The protein expression levels of GLI1 in glomerular mesangial cells (MC), endothelial cells (EC), podocytes (PC), renal tubular epithelial cells (RTEC), and mononuclear macrophages cells (RAW) *n* = 3; B) Immunofluorescence staining was used to detect the expression of GLI1 and PDGFRβ in the kidney of *db/m* and *db/db* mice (PDGFRβ, green fluorescence; GLI1, red fluorescence; Scale bar: 20 µm); C) The protein and mRNA expression levels of GLI1 in glomerular mesangial cells; D,E) The mRNA expression of GLI1 in mesangial cells; F,G) The protein expression levels of GLI1 in mesangial cells. *n* = 3; H) ChIP‐qPCR was used to detect the binding of GABP to the GLI1 promoter in mesangial cells; I) Dual luciferase reporter assay of transcription activation for GABPα and β on GLI1; J) Prediction of potential binding sites of GABP and GLI1 via JASPAR; K) Dual‐luciferase reporter assay to detect the predicted binding site of mutant GLI1 to GABP; L,M) The mRNA expression levels of PTCH1, VEGFC, VEGFD and Snail in mesangial cells. NG: normal mesangial cell; OV: normal mesangial cell with vector; OE: normal mesangial cell with GABPα/β‐overexpression lentivirus; HG: mesangial cell with 30 mM glucose; HKV: high glucose cultured mesangial cell with vector; HKD: high glucose cultured mesangial cell with GABPβ‐knockdown lentivirus. Data are expressed as mean  ±  s.e.m. Statistical significance was assessed using an unpaired t‐test (C, L, M, K) or one‐way ANOVA with Tukey's test (D, E, F, G, I, N) or two‐way ANOVA with Tukey's test. ^*^ *p* < 0.05, ^**^ *p* < 0.01, compared to NG, OV or HKV.

In the kidney, GLI1 was expressed in various types of renal parenchymal cell lines, including GMCs, RTECs, macrophage cells (RAWs), ECs, and PCs (**Figure** **6**A). The results showed that GLI1 colocalized with PDGFRβ in *db/m* and *db/db* mice, and the expression of GLI1 tended to increase in *db/db* mouse kidneys (Figure 6B). Furthermore, HG increased the expression of GLI1 in GMCs (Figure 6C). qPCR (Figure 6D‐E) and western blotting (Figure 6F‐G)showed that the expression of GLI1 was increased by GABP overexpression and reduced by GABP knockdown in GMCs. These results indicated that GABP positively regulates GLI1 expression in GMCs.

To further determine the regulatory mechanism of GABP on GLI1, we first performed ChIP assays and observed that GABPα was recruited to the promoter region of GLI1, and HG promoted the increase of GABPα and GLI1 binding (Figure 6H). The GLI1 promoter‐driven luciferase activity of the reporter plasmid dramatically increased when it was cotransfected with GABPα+GABPβ compared with an empty vector. In contrast, no significant change in luciferase activity was observed when GLI1 was cotransfected with GABPα or GABPβ alone, which may reflect the heterodimerization of the transfected GABP subunit with endogenous GABPβ or GABPα (Figure 6I). Next, the JASPAR database predicted two GABP‐binding sites in the GLI1 promoter sequence (Figure 6J). In addition, a mutant luciferase reporter plasmid was constructed, and three deletion mutant plasmids, GLI1 mutant 1 (−297 to −307), GLI1 mutant 2 (+1751 to +1761), and GLI1 1 and 2, were constructed. No change was observed in the activity of dual luciferase at site 1, whereas the activity at site 2 decreased, indicating that the mutation at 2 sites may be an effective GABP‐binding site (Figure 6K). These results suggest that GABP upregulates the transcriptional activity of GLI1 by binding to EBS sequences on the GLI1 promoter. In addition, GABP overexpression increased the mRNA level of the GLI1 target gene, whereas GABP knockdown decreased the mRNA level of the GLI1 target gene (Figure 6L,M), indicating that GABP can further affect the disease phenotype by regulating the expression of the GLI1 target gene through GLI1.

### 2.7 GABP Promotes Mesangial Cell Proliferation and ECM Deposition Through GLI1

**Figure 7:**
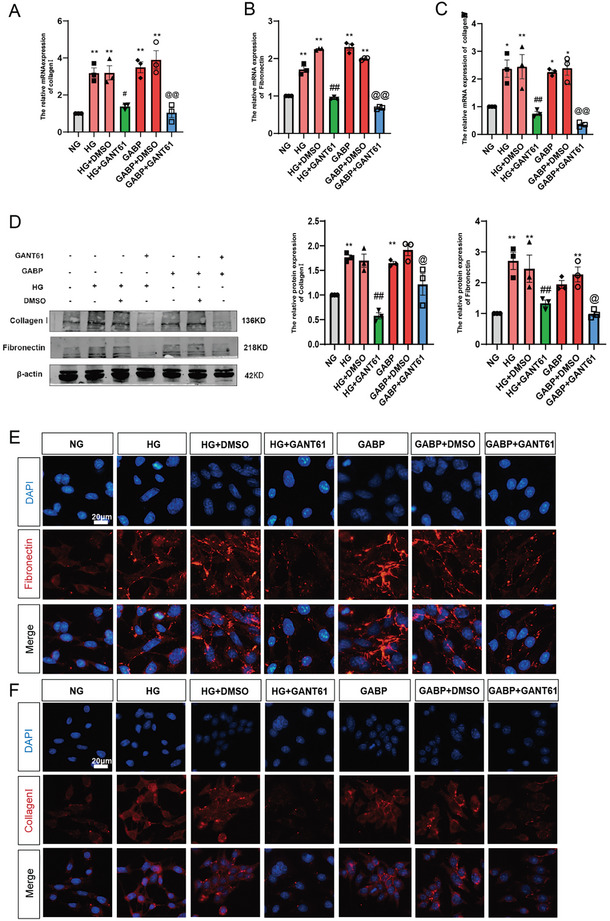
GABP Regulates Cell Proliferation and ECM by Inducing GLI1 Expression in Mesangial Cells. A–C) The mRNA expression levels of Collagen I, Fibronectin, and Collagen IV, *n* = 3; D) The protein expression levels of Collagen I and Fibronectin, *n* = 3; E,F) The accumulation of Fibronectin and Collagen I in cells was detected by immunofluorescence (DAPI, blue fluorescence; Fibronectin/Collagen I, red fluorescence; Scale bar: 20 µm). NG: normal mesangial cell group (5.56 mmol L^−1^ glucose concentration); HG: mesangial cell with 30 mM glucose group; HG+DMSO: high glucose cultured mesangial cell with DMSO control group; HG+GANT61: high glucose cultured mesangial cells with 10 µM GANT61 group; GABP: normal mesangial cells with GABPα/β‐overexpression lentivirus; GABP+DMSO: normal mesangial cells with GABPα/β‐overexpression lentivirus and DMSO group; GABP+GANT61: normal mesangial cells with GABPα/β‐overexpression lentivirus and 10 µM GANT61 group; Data are expressed as mean  ±  s.e.m. Statistical significance was assessed using one‐way ANOVA with Tukey's test, ^*^p < 0.05, ^**^p < 0.01, compared to the NG group, ^#^ *p*<0.05, ^##^ *p*<0.01, compared to the HG group, ^@^ *p* < 0.05, ^@@^ *p* < 0.01, compared to the GABP group.

To investigate whether the influence of GABP on the proliferation of GMCs is contingent upon GLI1, we utilized small interfering RNA to suppress GLI1 expression in GMCs with overexpressed GABP. The upregulation of mesangial cyclin resulting from GABP overexpression was markedly diminished following GLI1 interference (Figure S5A–C, Supporting Information). Inhibition of GLI1 function using a specific GLI inhibitor, GANT61, at a concentration of 10 µM for 24 h, led to a reduction in cell proliferation of glomerular mesangial cells (GMCs), as demonstrated by the CCK8 assay (Figure S5D,E, Supporting Information). We investigated the expression of cyclin following the administration of GANT61, and our findings indicated that this inhibitor significantly reduced cyclin expression (Figure S5F–H, Supporting Information). Quantitative qPCR (**Figure** **7**A–C), western blot analysis (Figure 7D), and immunofluorescence (Figure 7E,F) demonstrated that high glucose (HG) conditions and GABP overexpression resulted in ECM accumulation in GMCs. Furthermore, the GLI1 inhibitor GANT61 mitigated this accumulation. These results imply that GABP facilitates mesangial cell proliferation and ECM accumulation through the GLI1 pathway.

### 2.8 Inhibition of GLI1 Effectively Ameliorated GABP‐Mediated Renal Fibrosis in Diabetic Mice

**Figure 8:**
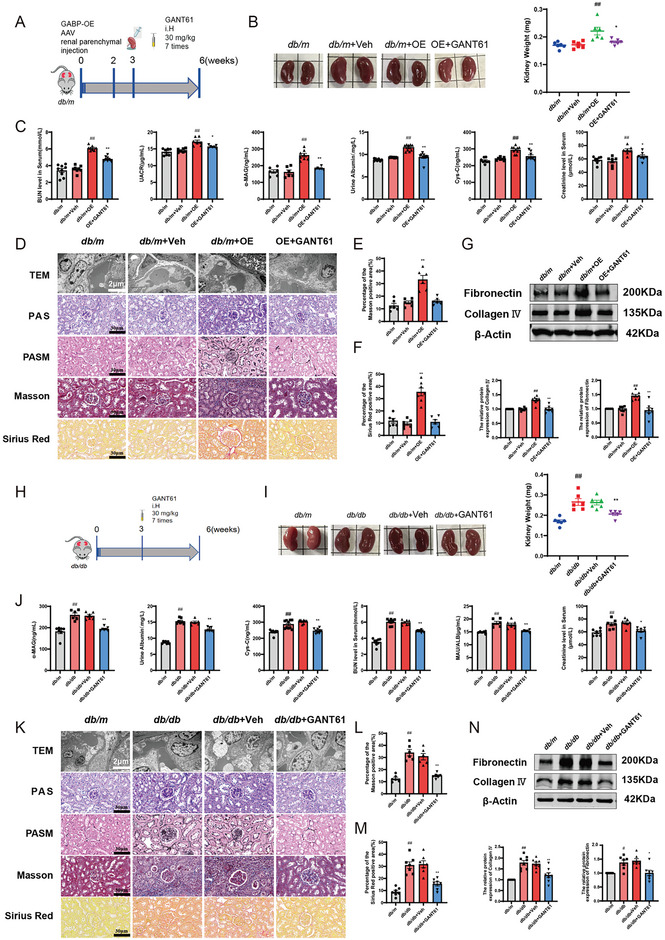
Inhibition of GLI1 Effectively Ameliorated GABP‐Mediated Renal Fibrosis in Diabetic Mice. A) Experimental procedure of GANT61 administration in *db/m*+OE mice; B) Kidney volume and weight of mice, *n* = 6; C) BUN, Cre, UACR, Cys‐C, α1‐MG and albumin of mice, *n* = 6; D) Transmission electron microscopy (Scale bar: 2 µm), PAS staining (Positive area: red), PASM staining (Positive area: black), Masson staining (Positive area: blue) and Sirius red staining (Positive area: red) of kidney tissue in mice (Scale bar: 20 µm); E,F) The quantitation of Masson and Sirius red postive staining areas, *n* = 6; G) The protein expression level of FN and Collagen I in mice kidney by western blot, *n* = 6; H) Experimental procedure of GANT61 administration in *db/db* mice; I) Kidney volume and weight of mice, *n* = 6; J) BUN, Cre, UACR, Cys‐C, α‐MG and albumin of mice, *n* = 6; K) Transmission electron microscopy (Scale bar: 2 µm), PAS staining (Positive area: red), PASM staining (Positive area: black), Masson staining (Positive area: blue) and Sirius red staining (Positive area: red) of kidney tissue in mice (Scale bar: 20 µm); L,M) The quantitation of Masson and Sirius red postive staining areas, *n* = 6; N) The protein expression level of FN and Collagen I in mice kidney by western blot, *n* = 6. *db/m*: normal control mice; *db/m*+veh: *db/m* mice with intra‐renal injection of vector; *db/m*+OE: d*b/m* mice with intra‐renal injection of GABPα/β overexpression adeno‐associated vins; OE+GANT61: *db/m*+OE mice with GANT61 (30 mg kg^−1^, once every two days for 3 weeks) administration; *db/db*: diabetic model mice; *db/db*+Veh: *db/db* mice with equivalent dose of the corn oil; *db/db*+GANT61: *db/db* mice with GANT61 (30 mg kg^−1^, once every two days for 3 weeks) administration. Data are expressed as mean  ±  s.e.m. Statistical significance was assessed using a one‐way ANOVA with Tukey's test. ^*^ *p* < 0.05, ^**^ *p* < 0.01, compared to OE+GANT61 or *db/db*+veh, ^#^ *p* < 0.05, ^##^ *p* < 0.01, compared to *db/m* or *db/db*.

To further elucidate the role of GLI1 in GABP‐mediated renal fibrosis in a murine model of diabetic nephropathy, we administered the GLI1 inhibitor GANT61 subcutaneously at a dosage of 30 mg kg^−1^ to both *db/m*‐OE and *db/db* mice (**Figure** **8**A,H). The findings indicated that the renal size of mice treated with GANT61 was significantly reduced compared to that of the *db/m*+OE and *db/db* groups (Figure 8B,I). Following the administration of GANT61, there was a notable reduction in the levels of renal function indicators, including creatinine (Cre), BUN, albumin, UACR, cystatin C (Cys‐C), and α‐MG, demonstrating significant improvement compared to the *db/m*+OE and *dbdb* groups (Figure 8C,J). Additionally, electron microscopy and morphological staining analyses corroborated that GLI1 inhibition substantially ameliorated the renal condition in mice with DN (Figure 8D–M). The expression of associated ECM proteins in mouse kidneys was assessed using western blotting and immunohistochemistry. The findings revealed that the expression levels of Fibronectin and Collagen‐IV proteins were significantly decreased following the administration of GANT61, in comparison to the *db/m*+OE and groups (Figure 8G‐N). These results suggest that GABP modulates renal fibrosis in diabetic mice via GLI1, and that the inhibition of GLI1 can attenuate renal fibrosis in mice with DN.

### 2.9 Machine Learning Approach Evaluates the Clinical Diagnostic Efficacy of GABP in Diabetic Nephropathy

**Figure 9:**
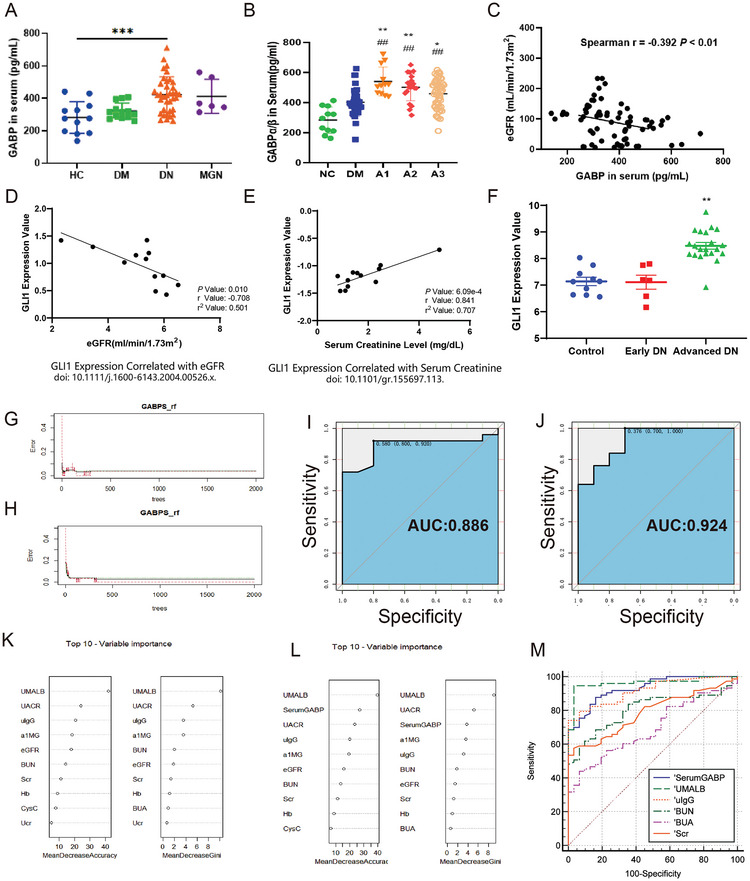
Machine Learning Approach Evaluates the Clinical Diagnostic Efficacy of GABP in Diabetic Nephropathy. A) The expression levels of GABP in human serum on healthy control volunteers (HC), type 2 diabetes without any kidney disease (DM), diabetic nephropathy (DN) and membranous glomerulonephritis (MGN) groups; B) The serum expression levels of GABP in different stage of diabetic nephropathy (A1: UACR < 30 mg g^−1^, A2: UACR 30–300 mg g^−1^, A3: UACR > 300 mg g^−1^); C) Correlation analysis between GABP expression in serum and estimated glomerular filtration rate (eGFR) of patient; D) Correlation analysis between GLI1 expression and eGFR of patient in the Nephroseq; E) Correlation analysis between GLI1 expression and serum creatinine of patient in the Nephroseq; F) The relative expression levels of GLI1 in normal nephrectomy samples adjacent to tumors (Control), different stage of diabetic nephropathy (Early DN: UACR between 30 and 300 mg g^−1^, eGFR >90 mL min^−1^/1.73 m^2^; advanced DN: UACR >300 mg g^−1^, eGFR <90 mL min^−1^/1.73 m^2^), Data sourced from GSE142025 in the GEO; G,H) The relationship between the model error rate and the number of decision trees without GABP. The red line represents the error rate of DM prediction, the green line represents the error rate of DN prediction, and the black line represents the error rate of out‐of‐pocket samples. I,J) ROC curve of (G, H) model. K,L) The top 10 important variables of (G, H) model. M) ROC curve of several renal function indexes, *n* = 120. Data are expressed as mean  ±  s.e.m. Statistical significance was assessed using one‐way ANOVA with Tukey's test. ^*^ *p* < 0.05, ^**^ *p* < 0.01, ^***^ *p* < 0.001, compared to the HC or Control group, ^##^ *p* < 0.01, compared to the DM group.

Finally, serum samples were collected from healthy control volunteers (HC), DN, type 2 diabetes without kidney disease (DM), and membranous glomerulonephritis (MGN). Compared with the HC group, the expression of GABP protein in the serum of patients with DN was significantly higher, but there was no significant difference in patients with MGN (**Figure** **9**A; basic clinical data listed in Table S2, Supporting Information). At the same time, compared to the DM group, GABP increased more significantly in the early stages (A1 and A2) of DN (Figure 9B), highlighting the advantage of GABP as a biomarker of early DN. Correlation analysis showed that the protein expression levels were negatively correlated with eGFR (Figure 9C). The expression level of GLI1 negatively correlated with eGFR and positively correlated with serum creatinine levels. Analysis of clinical data in the database showed that compared with the control group, GLI1 did not increase significantly in the early stage of DN but only showed statistical significance in the advanced stage (Figure 9D–F). Based on the expression of GABP and GLI1 and the results of the correlation analysis, we believe that GABP in serum has high clinical application value. Therefore, we expanded the patient population and further analyzed GABP in serum. The random forest package in R software was used for calculations, and 26 indicators related to renal function were included as covariates. The generation model error rate and number of decision tree diagrams showed that GABP reduced the benefit model error rate, improved the stability of the model, and decreased model calculations (Figure 9G,H). The Receiver Operator Characteristic (ROC) curve of the models showed that the AUC value was 0.886 (Figure 9I) when GABP was not included but increased to 0.924 (Figure 9J) after the inclusion of GABP. After GABP was included, its importance ranked very high and was higher than that of certain clinical renal function indicators (Figure 9K,L). These results indicated that GABP is beneficial for improving the disease prediction ability of the DN model. ROC curve analysis showed that the Area Under Curve (AUC) of serum GABP was 0.931, which was superior to that of Scr and BUN in the diagnosis of DN and comparable to that of urinary microalbumin (Figure 9M). In conclusion, serum GABP levels have a high clinical value in the diagnosis of DN.

## 3 Discussion

In this study, we used proteomics to identify GABP, a transcription factor closely related to DN renal fibrosis, in the kidneys of diabetic mice with renal fibrosis symptoms. We further explored the potential mechanism by which GABP affects the DN renal fibrosis process through in vivo and in vitro biological methods, such as transcriptomics, and determined the role of GABP in the early diagnosis of DN through retrospective clinical analysis.

This study offers two main contributions. First, through proteomics, we observed that GABP plays an important role in DN and explored its function and potential mechanisms in DN renal fibrosis for the first time. Renal fibrosis is the ultimate pathological outcome of most chronic progressive kidney diseases, including DN.^[^ ^21^ ^]^ Pathological features of the glomeruli during DN progression include mesangial cell proliferation, mesangial matrix hyperplasia, basal membrane thickening, podocyte loss, and glomerulosclerosis.^[^ ^22^, ^23^, ^24^ ^]^ However, there is still a lack of a comprehensive understanding of the pathogenesis of renal fibrosis, which hinders the development of effective treatments and early diagnostic biomarkers. DN is characterized in its early stages by glomerular and mesangial hypertrophy, which is caused by GMC proliferation and ECM expression.^[^ ^25^ ^]^ Increasing evidence suggests that the genes that mediate the pathogenesis of DN renal fibrosis are regulated by transcription factor binding.^[^ ^26^, ^27^ ^]^ In the current study, TMT proteomic techniques were used to detect the altered proteins in the kidneys of mice with DN. GABP expression changed significantly in GO terms for DNA‐binding transcription factor activity. As a transcription factor involved in the occurrence and development of many diseases. In human hematopoiesis, GABP is involved in maintaining hematopoietic stem cells and differentiation of the bone marrow and lymphoid lineage and plays an important role in the proliferation of bone marrow progenitor cells.^[^ ^28^ ^]^ In the brain, GABP is believed to be involved in forming neuromuscular connections by regulating the transcription of synapse‐specific genes necessary for synaptic differentiation in vivo.^[^ ^29^ ^]^ However, research in the field of nephrology is still in its infancy, and studies on its cellular regulatory function and role in renal fibrosis in DN have not been reported. The GABP is composed of one Ets‐related GABPα subunit and an ankyrin repeat‐containing GABPβ subunit.^[^ ^30^ ^]^ The combined action of multiple transcription factors is an important mechanism that allows GABP to regulate lineage‐restricted target genes.^[^ ^31^, ^32^ ^]^ The change in GABP expression levels in the serum of mice of different ages and their correlation with UACR in mice demonstrate the potential of GABP as an early biomarker of renal function damage in diabetic nephropathy. In contrast, we observed that in *db/db* mice of older age (24 weeks), the serum expression of GABP did not exhibit a strong correlation with previously anticipated renal function, suggesting that during the advanced stages of diabetic nephropathy, GABP, which functions as a transcription factor, is disrupted and loses its balance in regulating the renal fibrosis process. Consistent with previous studies,^[^ ^12^ ^]^ we observed that GABP was mainly expressed in glomerular GMCs and podocyte nuclei in the kidneys. Moreover, we observed that GABP is necessary and sufficient to drive quiescent cells into the cell cycle.^[^ ^19^ ^]^ We confirmed through in vivo and in vitro experiments that GABP may be associated with DN‐related GMC proliferation, renal fibrosis, and renal function changes.

Second, we explored the mechanism of GABP‐mediated GLI1 regulation in DN renal fibrosis using biological techniques, such as transcriptomics. Through bioinformatics analyses, such as IPA, it was observed that interference with GABP expression in mouse kidneys can significantly affect the regulation of fibrosis‐related pathways, affecting the expression of multiple downstream genes related to fibrosis. Among them, the downstream differentially expressed gene GLI1 had the greatest correlation with GABP expression and was most closely related to fibrosis and proliferation‐related diseases. Simultaneously, our biological experiments, such as ChIP‐qPCR and dual luciferase assays, confirmed the interaction between GABP and the GLI1 promoter. GABP positively regulates the transcription of GLI1, and high glucose levels promote the binding of GABP to the GLI1 promoter, verifying the results of the bioinformatics analysis. GLI1, an end effector of the sonic hedgehog pathway, control cells differentiation and morphogenesis in many tissues and species.^[^ ^33^ ^]^ SHH induces the expression of α‐smooth muscle actin, junction protein, FN, and collagen I by activating GLI1 in renal fibroblasts.^[^ ^34^, ^35^, ^36^ ^]^ Our results showed that interfering with GLI1 expression could reduce the collagen accumulation and fibrin reduction induced by GABP. GABP regulates GLI1 target genes and affects DN renal fibrosis through GLI1.

Finally, we discussed the clinical applications of GABP and GLI1. We checked the expression of GABP in the serum of patients and that of GLI1 in the GEO database. We observed that compared to the NC group, the expression of GABP in DM and DN was significantly higher, but there was no significant change in the expression of GABP in other nephropathies (such as Membranous Glomerulonephritis, MGN). Compared to the DM group, GABP increased more significantly in the early stages (G1 and G2) of DN. GABP expression is closely related to renal function in DN, which increases with a decrease in eGFR. These results suggest that GABP may be a specific biomarker of early DN. The role of GLI1 is reflected in the stage of advanced DN. Machine learning seeks to predict outcomes or classify patient characteristics by identifying patterns in large data sets.^[^ ^37^ ^]^ They are increasingly being applied to predict early diabetes and its complications. Several studies have reported predictive models for diabetic complications and glycemia.^[^ ^38^, ^39^ ^]^ Random Forest^[^ ^40^ ^]^ is an integrated machine‐learning algorithm that can be used for classification and regression tasks and can give importance to the ranking of variables in the model. We observed that serum GABP plays an important role in early DN clinical prediction models, and its diagnostic performance is comparable to that of traditional DN biomarkers such as UMALB and uIgG. Simultaneously, the presence of the GABP variables improved the stability and performance of the prediction model. However, this was a retrospective study, and the observation time for the patients was relatively short. In the future, we plan to design more long‐term clinical trials to explore the clinical value of GABP based on the current observations.

## 4 Experimental Section

### Experimental Animals—Diabetic Mouse Model

For proteomics analysis, C57BL/6J mice were treated to two groups (n ≥ 6/group): C57 group (normal control mice) and STZ group (STZ diabetic model mice). High‐fat diet combined with streptozotocin (STZ, S0130, Sigma, USA) injection method was used to induce type 2 diabetic mice model (T2DM) as described before. Briefly, after 8 weeks high‐fat diet feeding, STZ group mice were treated with STZ intraperitoneal injection (100 mg kg^−1^) which was dissolved in 0.1 M citrate buffer, pH 4.5 with the final concentration of 1% and C57 group mice received the same volume solvent instead. The mice were considered diabetic when the fasting blood glucose (FBG) levels above 16.7 mM. Mice were fed for another 8 weeks and then sacrificed. Blood and kidneys were collected for further study.

### Experimental Animals—Renal Parenchymal Injection

The steps of targeted injection into the renal parenchyma through the back as follows: first, Anesthetize (1.5‐2%, Orbiepharm, China) the mouse with isoflurane, then position the mouse prone on the operating table. And locate the approximate position of the kidney from the back, shave the mouse's back bilaterally, make a 1.5 cm incision on the left back, and with both hands, extract the left kidney and gently separate the fat. Then, clamp the renal hilum to prevent the virus solution from flowing down to the bladder. Use a 30 G needle to puncture the upper, middle, and lower poles of the left kidney, inject 50 µL of liquid (containing 5 × 10 ^ 10 µg viral particle genome copies or saline) into the renal pelvis, and quickly inject the liquid into the renal cortex for at least 30 s before slowly withdrawing the needle; the key to puncturing the renal cortex without penetrating the kidney lies in comparing the puncture needle with the mouse kidney and marking it on the needle. Finally, observe for 5 min, and remove the microvascular hemostatic clamp and suture the incision in two layers.

### Experimental Animals—GABPα/β Mouse Model

To explore the effect of GABP on renal fibrosis in diabetes nephropathy (DN), *db/m* mice were randomly divided into a normal control group (*db/m*), empty vector group (*db/m*+veh), and GABPα/β overexpression group (*db/m*+OE); *db/db* mice were randomly divided into a model control group (*db/db*), empty vector group (*db/db*+veh), and GABPβ knockdown group (*db/db*+KD), injected with physiological saline (control group), empty vector AAV (empty vector group), and GABPα/β overexpression or GABPβ knockdown of AAV into the renal parenchyma of mice at 12 weeks of age. The amount of AAV injected was 5 × 10^10^µg per animal. Animals were sacrificed after 1 month of regular feeding (n ≥ 6/group). Next, mouse kidneys are removed for transcriptomic analysis.

### Experimental Animals—GANT61 Mouse Model

To elucidate the role of GLI1 in GABP‐mediated renal fibrosis in a murine model of diabetic nephropathy. First, *db/m* mice were randomly divided into a normal control group (*db/m*), GABPα/β overexpression empty vector group (*db/m*+veh), GABPα/β overexpression group (*db/m*+OE) and GANT61 administration in *db/m*+OE (OE+GANT61) group; *db/db* mice were randomly divided into a model control group (*db/db*), GANT61 empty vector group (*db/db*+veh), and GANT61 administration in *db/db* group (*db/db*+GANT61). Then, the GLI1 inhibitor GANT61 (421 610, Aladdin, China) was administered at 30 mg kg^−1^ per mouse, once every two days for 3 weeks, by subcutaneous injection to both the OE+GANT61 group and the *db/db*+GANT61 group. GANT61 was dissolved in corn oil (G2408351, Aladdin, China), while the *db/m*+OE group and the *db/db*+veh group mice received an equivalent dose of the solvent as a control. Animals were sacrificed after 1 month of regular feeding (n ≥ 6/group). Next, mouse kidneys are removed for transcriptomic analysis.

SPF grade male C57BL/6J wild‐type mice, provided by the Laboratory Animal Center of Xuzhou Medical University (Xuzhou, China). Male C57BLKS/J background Lep^db^/Lep^db^ (*db/db*) mice (7 weeks of age, 30–35 g) and their litter control Lep^db^/Lep^m^ (*db/m*) mice were obtained from the Model Animal Research Center of Nanjing University (Nanjing, China). Before and during the experiment, all animals were kept in the same cage, with free access to water and food. All experimental procedures were approved by the Animal Ethics Committee of Xuzhou Medical University (Ethics No: 201812W008; approved by December 2018) and followed the Guiding Principles for the Care and Use of Laboratory Animals of Xuzhou Medical University.

### Renal Function Assessment

The FBG of mice were tested by blood glucose meter (Johnson, CA, USA) through the tail veins. Mice urine was taken before sacrificed and after 3000 rpm centrifugation at 4 °C for 10 min, urine albumin, creatinine and micro‐albumin (MAU) were measured as the manufacture instructions of the ELISA kits (LP‐M03268; LP‐M01553; LP‐M03270, Lanpai Biology, Shanghai, China), and calculate the level of UACR from the ratio of MAU to Cre. Mice serum was collected from the blood after 3000 rpm centrifugation for 15 min at room temperature and blood Cre together with blood urea nitrogen (BUN) were determined by assay kits (C011‐2; C013‐2, Jiancheng Bioengineering Institute, Nanjing, China).

### Renal Morphological Assessment

Tissue sections of 4 µm thickness were prepared from paraffinembedded kidney tissue and then stained with PAS, HE, Masson, and Sirius red after deparaffinization to assess kidney morphology, glycogen deposition, and collagen accumulation. The kits were purchased from Solarbio (Beijing, China). Images were taken randomly and blindly under a microscope (OLYMPUS, Japan).

### TMT Proteomics

Proteomics analysis were performed as described before.^[^ ^41^, ^42^ ^]^ Briefly, 6 normal control mice kidneys (C57) and 6 diabetic mice kidneys (STZ) were randomly divided into two groups respectively (totally 4 samples). The kidney samples were homogenized and lysed use SDT method. After FASP digestion, the peptide fragments (100 µg) from each sample were labeled as the instructions of the TMT kit (Thermo Fisher Scientific, TMT 6/10 plex Isobaric Label Reagent) and TMT‐labeled digest samples were fractionated use a pierce high pH reversed‐phase fractionation kit (Thermo Fisher Scientific) into 15 fractions and then subjected to mass spectrometry. LC‐MS/MS analysis was performed on a Q Exactive mass spectrometer coupled with Easy nLC (Thermo Fisher Scientific) for 90 min. The original data were analyzed via Mascot2.2 and Proteome Discoverer1.4 to identify and quantitate the proteins.

Blast2GO (https://www.blast2go.com/) was used to perform GO annotation of the target protein collection. The Fisher's exact test was used to compare the distribution of each GO class in the target protein set and the overall protein set, and the GO annotated enrichment analysis was performed on the target protein set. The symbol of mouse genes under DNA‐binding transcription factor activity GO term were queried through the MGI database (https://www.informatics.jax.org/). Based on the information in the STRING database (http://string‐db.org/), the interaction relationship between the target proteins was found, and the interaction network was generated and analyzed by using Cytoscape software (version 3.2.1).

### RNA‐Seq Transcriptomic Analysis

As previously described,^[^ ^43^ ^]^ total RNA was extracted from the kidneys of mice in the *db/db+*veh, *db/db+*KD, *db/m+*veh, and *db/m+*OE groups using Trizol Reagent (Shanghai, China). The NanoDrop One Microvolume UV‐Vis spectrophotometer (Thermo, USA) was employed to determine the concentration and purity of the total RNA of the different samples. The total RNA samples were then submitted to Shanghai Bioprofile Co., Ltd. (Shanghai, China) for preparation and construction of the mRNA library, followed by transcriptomic sequencing on the HiSeq X Ten System (Illumina, San Diego, CA, USA). Sequencing data generally contained a number of connectors and low‐quality reads. The Cutadapt (v2.7) software was used to filter the sequencing data to obtain high quality sequence (Clean Data) for further analysis. Use HISAT2 (http://ccb.jhu.edu/software/hisat2/index.shtml) software to map the clean reads onto the reference genome. HTSeq (Python package, v2.0.5) was used to calculate the read count mapped to each gene as the raw expression of the gene, and FPKM (Fragments Per Kilo bases per Million fragments) was used to normalize the expression levels.

DESeq (Bioconductor Package, v1.8.3) was used to analyze the difference in gene expression, and the conditions for screening the differentially expressed genes (DEG) were as follows: |log_2_FoldChange| > 1, *p* value <0.05. Bidirectional clustering analysis was performed on the union and samples of differentially differentiated genes of all comparison groups using the Pheatmap (R package, v1.0.12). The volcano map of differentially expressed genes was plotted using the ggplot2 (R package, v3.5.0).

Enrichment classical pathway analysis was performed using IPA (Ingenuity Pathway Analysis, QIAGEN) software application. Use the VarElect database (https://varelect.genecards.org/) to prioritize the association of differential genes with disease or phenotype. The GEPIA database (http://gepia.cancer‐pku.cn/) was used to analyze the correlation between differential genes and GABP.

### Immunohistochemistry, IHC

Fixed kidney section of a 4 µm thickness were deparaffinized in xylene and rehydrated in a graded series of alcohols. Subsequently, the sections were incubated with 3% H_2_O_2_ for 10 min to eliminate endogenous peroxidase activity. After pepsin antigen retrieval for 30 min, the sections were washed with PBS three times for 3 min each and then blocked with 2% BSA for 0.5 h at room temperature. The sections were incubated with primary antibody (1:200) at 4 °C overnight. Sections were stained using a polymer HRP detection system (ZSGB‐BIO, Beijing, China) and visualized using a DAB detection kit (Vector Laboratories Inc, Burlingame, CA, United States). After conventional dewatering and neutral balsammounting, photographs were taken in random fields using an Olympus BX43F microscope (OLYMPUS, Japan).

### Immunofluorescence, IF

Kidney samples were fixed in 4% paraformaldehyde and embedded in paraffin. Sections of 4 µm thickness were cut perpendicularly along the long axis of the kidney and they were used for immunofluorescence analysis. The sections were deparaffinized in xylene, hydrated in graded alcohol and water, and placed in 3% H_2_O_2_ for 10 min to eliminate endogenous peroxidase activity. After incubating the sections for 30 min with pepsin, they were washed three times in PBS, and the samples were blocked at room temperature with 2% BSA for 0.5 h. The sections were incubated with a primary antibody at 4 °C overnight and then with a secondary antibody at 37 °C for 1 h. Sections were stained with DAPI (Beyotime, Nantong, China), and observed with an Olympus BX43F fluorescence microscope (Tokyo, Japan) or laser scanning confocal microscope (Leica STELLARIS 5, Germany). Cells in the 12‐well plates were washed three times with cold PBS and fixed at −20 °C for 20 min with cold methanol. After washing with PBS three times, the cells were sealed with 2% BSA at room temperature for 1 h, and then the same procedure was performed as described above.

### Cell Culture and Transfections

Mouse glomerular mesangial cells (GMCs) SV40 MES 13 was purchased from the Beijing Golden Amethyst Biomedical Technology Co., Ltd (Chinese Academy of Medical Sciences). Mouse renal tubular epithelial cells (MRTEC) were purchased from Shanghai Lianmai Biotechnology Co., Ltd; Mouse macrophages (RAW 264.7 cells) were purchased from Bohui Biotechnology (Guangzhou) Co., Ltd; Mouse endothelial cells (EC) were purchased from Procell Life Science&Technology Co., Ltd.; Mouse podocytes (MP) were purchased from Shanghai Qingqi Biotechnology Development Co., Ltd. GMCs were cultured in DMEM medium containing 5.56 mmol L^−1^ glucose (normal glucose [NG]; 31600034, GIBCO by Life Technologies, USA); supplemented with 14 mM HEPES, 5% fetal bovine serum (10099‐141, GIBCO by Life Technologies, USA), and 1% penicillin and streptomycin (100X; P11‐010, Beyotime Institute of Biotechnology, Nantong, China); and grown in a 5% CO_2_ humidified atmosphere at 37 °C. The cells were passaged at 80–90% confluency.

Transfection of GABP lentivirus in mesangial cells. The fusion degree of normal cultured mouse glomerular mesangial cells reaches 30–40%, and lentiviral infection is performed. Dilute the mother liquor of lentivirus which contains GFP flag with serum‐free culture medium to a titer of 1 × 10^8^ TU/ml virus solution, add the virus amount at MOI = 30, and after 8 h of infection with serum‐free culture medium, replace it with complete culture medium. Continue to culture for 3 days, observe the fluorescence intensity under a fluorescence microscope to determine the transfection efficiency (more than 70% is considered successful transfection), and detect the expression of GABPα and GABPβ through qPCR, Western blotting, and IF. The GABP α/β overexpression experiment was divided into three groups: normal group (5.56mmol L^−1^, NG), normal mesangial cells with vector group (NG+veh, OV), and normal mesangial cells with GABPα/β overexpression lentivirus group (NG+GABP α/β‐OE, OE). The GABPβ knockdown group was divided into four groups: normal group (5.56 mmol/L, NG), mesangial cells with 30 mM glucose group (HG), high glucose cultured mesangial cells with vector group (HG+Veh, HKV), and high glucose cultured mesangial cells with GABPβ knockdown lentivirus group (HG+GABP‐KD, HKD).

Co‐transfection of GABP and GLI1 in GMCs. When the confluency of GMCs in normal culture reached 40%–50%, GABP overexpression lentivirus infection was performed. Observe the fluorescence intensity under the fluorescence microscope to judge the transfection efficiency, if it reaches 70%, the transfection is successful. Dilute the appropriate amount of plasmid with jet PRIME, mix well, and let it stand for 10 min, then add jet Reagent proportionally to prepare the master mix. Add the GLI1 knockdown plasmid‐Reagent complex to the cells and incubate the cells at 37 °C for 2 days. The experiment was divided into 6 groups: NG: normal group (5.56 mmol L^−1^ glucose concentration), HG: high glucose group (30 mmol L^−1^ glucose concentration), OV: GABP overexpression empty vector virus group, HG+shGLI1: high glucose knockdown GLI1 group, GABP: GABP overexpression group, GABP+shGLI1: knockdown of GLI1 in stably overexpressing GABP cell lines.

In addition to interfering with GLI1 mRNA expression, the protein expression of GLI1 with GANT61 was further inhibited, a specific inhibitor of GLI1. First, the cytotoxicity of GANT61 to GMCs was measured by CCK8, and selected a concentration of 10 µM GANT61 for subsequent experimental studies. Next, 10 µM of GANT61 was administered to GABP overexpressing mesangial cells for 24 h, and qPCR, Western blotting, and IF were used to detect changes in the functions of GMCs such as cyclins, cell proliferation, and fibrosis. The experiment was divided into 7 groups: NG: normal group (5.56 mmol L^−1^ glucose concentration), HG: high glucose group (30 mmol/L glucose concentration), HG+DMSO: high glucose add DMSO control group, HG+GANT61: high glucose add 10 µM GANT61 group, GABP: GABP overexpression group, GABP+DMSO: the group of add DMSO in stably overexpressing GABP cell lines, GABP+GANT61: the group of add 10 µM GANT61 in stably overexpressing GABP cell lines.

### Flow Cytometry

The cells are digested using trypsin, centrifuged at 1000 g for 5 min, the supernatant is discarded, and the cells are collected. The cells are then resuspended in pre‐cooled 70% ethanol for fixation and incubated overnight at 4 °C. The following day, 1% FBS is added and thoroughly mixed, followed by centrifugation at 1000 g for 5 min to remove ≈70% of the ethanol. A mixture of PI (diluted 1:50) and RnaseA (diluted 1:100) is prepared. Subsequently, 300 µL of PI is added and the cells are incubated at 37 °C for 30 min. No washing step is necessary; simply mix the cells thoroughly. After filtering the sample through a 200‐mesh cell strainer, it is transferred to a 5 mL flow cytometry tube. Proceed with flow cytometry (BD FACSCanto II, America) detection by loading samples at a low speed and recording a total of 20000 target cells. Finally, collect and analyze the data.

### Cell Counting Kit‐8 (CCK‐8) Assay

To prepare an equal‐density cell suspension, the cells were collected and plated in 96‐well plates containing 100 µL media at a density of 5 × 10^3^ cells per well. Cells were serum starved for 24 h after adherence, followed by different treatments. The cells were then incubated with 10 µL CCK‐8 (Dojindo Laboratories, Kumamoto, Japan) for 1h. A microplate reader (BioTek, USA) was used to determine optical density (OD) at 450nm. The experiment was repeated three times to determine the mean OD value.

### Quantitative Real‐Time PCR, qPCR

RNA was isolated from the samples using RNA isolator Total RNA Extraction Reagent (R401‐01, Vazyme). RNA was reverse transcribed to complementary DNA using HiScript II Q RT SuperMix (R223‐01, Vazyme). Primers were designed and synthesized by Sangon Biotech (Shanghai, China). The data of qPCR were acquired by Roche LightCycler 480 II/96 software. The primers used are listed in Table S3 (Supporting Information).

### Western Blotting

Total lysate protein levels were quantified using a BCA Protein Assay kit (Thermo, USA) according to the manufacturer's protocol. Equal amounts of protein were electrophoresed on an 8–12% sodium dodecyl sulfate‐polyacrylamide gel, and the proteins were electrophoretically transferred onto nitrocellulose transfer membranes (Merck, Germany). The membranes were blocked using 3% bovine serum albumin (Solarbio, China) for 1 h at room temperature, incubated with primary antibodies overnight at 4 °C. Membranes, washed three times with PBST for 5 min and then incubated with IRDye 800CW goat anti‐mouse IgG (1:20 000 dilution) or goat anti‐rabbit IgG (1:20 000 dilution) for 1h. The membrane was scanned using Odyssey Sa (Li‐Cor, USA) in Image Studio version 5.2.5, and analyzed using Image J.

### ChIP‐qPCR

ChIP assays were performed in GMCs of the normal glucose (NG) and high glucose (HG) groups. Then, 8×10^6^ cells were cross‐linked using 8 mL 1% formaldehyde in PBS for 10 min at room temperature, washed with cold PBS, and resuspension in 250 µL of ChIP lysis buffer (1% SDS, 10 mM EDTA, 50 mM Tris‐HCl, pH8.0 and protease inhibitor cocktails) through sonication (30% maximum output for 15s with 3 min pause, four cycles) in an ice bath. A 5% portion of the lysate was used for extraction of total DNA for input gene quantification, and ChIP assays were performed with a Magnetic ChIP Kit (P‐2026, Epigentek) according to the manufacturer's instructions. The cells were lysed, and the chromatin was mainly fragmented to 200–1000 bp by sonication. DNA/protein complexes were precipitated by incubation with 0.8 g of antibodies against GABPΑ (25142‐1‐AP, Proteintech) or IgG, and then incubated with protein A/G magnetic beads for 2h. After the reversal of protein DNA cross‐linking, the DNA was purified, and the abundance of the GABPα and GLI1 promoters was analyzed by qPCR. qPCR analysis was performed using primers spanning the two predicted GLI1 regions within the promoter of the murine GABPα gene. GAPDH in DNA prior to immunoprecipitation was amplified as a control. Primers were listed below:

GLI1‐forward primer: CCCCTCTCTAGCTTCTATCCACCCAG,

GLI1‐reverse primer: TTTCTCGCTGTTGCCACCCG;

GLI1‐forward primer: GGGTCAGCCTGGACTACTGAGTAAC,

GLI1‐reverse primer: GGGTCAGCCTGGACTACTGAGTAAC.

### Dual‐Luciferase Reporter Assay

The WT and site‐mutated promoter sequences of the murine GLI1 gene were synthesized by the Probe Gene Company and subcloned into the pGL3‐Basic vector using MluI and HindIII recognition sites. All constructs were confirmed by automatic DNA sequencing. HEK293T cells were seeded in 96‐well plates and transfected with a firefly reporter vector (0.2 mg) and Renilla reporter vector (0.01 mg), together with other expression plasmids (0.2 mg) using GenXPIII (ProbeGene CB043) transfection reagent when cells reached up to 60% confluence. After transfection for 36 h, the ratio of firefly and Renilla luciferase reading was calculated and fluorescence values were detected using the Dual‐Luciferase Reporter Assay System (Promega E1901).

### Antibodies

The following antibodies were used: GABPα (21542‐1‐AP, Proteintech, WB: 1:2000, IHC: 1:50, IF/ICC: 1:200), GABPβ (12597‐1‐AP, Proteintech, WB: 1:1000, IHC: 1:50, IF/ICC: 1:500), GLI1 (66905‐1‐Ig, Proteintech, WB: 1:5000, IF/ICC: 1:400) and Fibronectin (15613‐1‐AP, Proteintech, WB: 1:2000, IHC: 1:2000, IF/ICC: 1:50) for Western blotting, IHC and IF staining; β‐actin (AP0060, Bioworld, WB:1:5000, IF: 1:500), Cyclin D1 (26939‐1‐AP, Proteintech, WB: 1:5000, IHC: 1:750, IF/ICC: 1:400), Cyclin E1 (11554‐1‐AP, Proteintech, WB: 1:500, IHC: 1:400) for Western blotting; Cyclin D1(A11022, ABclonal, WB: 1:500, IHC: 1:50, IF/ICC: 1:50), Cyclin E (120039, absin, WB: 1:500, IHC‐P: 1:100, IHC‐F: 1:100, IF: 1:100), PCNA (13110, Cell Signaling Technology, WB: 1:1000, IHC: 1:4000, IF: 1:400), KI67 (AF0198, Affinity, WB: 1:500, IHC: 1:50, IF/ICC: 1:100) and PDGF Receptor β (#3169, CST, WB: 1:1000, IHC: 1:50, IF: 1:100) for IF staining; COL1A1 (67288‐1‐Ig, Proteintech, WB: 1:5000, IHC: 1:2500, IF:1:200) for IF staining and Western blotting. For IF staining, the following secondary antibodies: Alexa Fluor 488 goat anti‐rabbit IgG (AB150077, Abcam, IF: 1:200), Alexa Fluor 488 donkey anti‐mouse IgG (AB150109, Abcam, IF: 1:200), Alexa Fluor 555 donkey anti‐rabbit IgG (AB150074, Abcam, IF: 1:200), and Alexa Fluor 594 donkey anti‐mouse IgG (AB150108, Abcam, IF:1:200). For Western blotting, secondary antibodies IRDye 800CW goat anti‐rabbit IgG (926‐32211, LI‐COR, WB: 1:5000) and IRDye 800CW goat anti‐mouse IgG (926‐32210, LI‐COR, WB: 1:5000) were used.

### Clinical Data

Patients with biopsy‐proven proteinuric kidney diseases, including diabetic nephropathy (DN), membranous glomerulonephritis (MGN), hospitalized in the Department of Nephrology, the Affiliated Hospital of Xuzhou Medical University, between September 2019 and August 2023 were retrospectively assessed. The specificity of GABP as a biomarker in DN patients was assessed using MGN patients. Patients with type 2 diabetes without any kidney disease (DM) and healthy control volunteers (HC) were evaluated retrospectively. The differentiation of GABP as a biomarker in DN was assessed using DM and HC. In this study, 48 patients with DM, 124 patients with DN, 15 patients with MGN, and 15 volunteers with HC who had complete clinical data, and retained urine as well as blood samples during the hospitalization period were enrolled.

Patients in DN group and DM group were used to establish a binary machine learning prediction model. Randomforest (R package, v4.7‐1.1) and caret (R package, v6.0‐94) are used to establish the machine learning model in R 4.2.3 and RStudio. The machine learning model is built using randomForest (R package, v4.7‐1.1) and caret (R package, v6.0‐94). VIM (R package, v6.2.2) was used to check the missing value of data, and random forest method was used to interpolate the variables with missing value less than 30%, and the variables with missing value more than 30% were deleted. The performance of the model was evaluated and ROC curves were drawn using pROC (R package, v1.18.4). The comparison of ROC curves between multiple univariates was performed using medcalc statistical software (Version 19.7.4). The correlation between GABP and EGFR was analyzed by Spearman method in GraphPad Prism 8.3.0.

This study was approved by the Ethics Committee of the Affiliated Hospital of Xuzhou Medical University (approval number XYFY2019‐KL222‐01) and this study was performed in accordance with the ethical standards as laid down in the 1964 Declaration of Helsinki and its later amendments. Informed consent was obtained from all patients to be included in the study.

### Statistical Analyses

Data are expressed as mean ± SD or SEM. An unpaired *t*‐test was used to analyze data between 2 groups. One‐way or 2‐way ANOVA with Tukey's multiple comparisons test was used when more than 2 groups were present, as indicated. All experiments were repeated at least 3 times, and representative experiments are shown. All tests were two‐tailed, and *p* < 0.05 was considered statistically significant. Comparison between groups was performed using GraphPad Prism 8.3.0 or SPSS 26.0.

## Conflict of Interest

The authors declare no conflict of interest.

## Author Contributions

L.D, S.L, Y.L contributed equally to this project. The experiments were designed by Q.L., L.D., and X.Y. The experiments were performed by L.D., X.Y., Y.L., Y.H., T.Y., D.R., S.L., Q.Y., J.M., and J.Z. L.D., X.Y., and Y.L. analyzed the data and wrote the manuscript.

## Supporting information



Supporting Information

## Data Availability

The data that support the findings of this study are available from the corresponding author upon reasonable request.
