# Supplementary material for: GABP Promotes Mesangial Cell Proliferation and Renal Fibrosis Through GLI1 in Diabetic Nephropathy
Source: Adv Sci (Weinh). 2025 Feb 22;12(15):2407462. doi: 10.1002/advs.202407462 (PMC12005803; doi:10.1002/advs.202407462)
Supplement: Supplementary file 1 — Supporting Information [file ADVS-12-2407462-s001.docx]

Suppoting Information

**GABP Promotes Mesangial Cell Proliferation and Renal Fibrosis through GLI1 in Diabetic Nephropathy**

#### Lei Du^a#^, Sijie Liu^a#^, Yinfei Lu^a#^, Dongxue Ren^a^, Xiujuan Yu^a^, Yue Hu^a^, Tingting Yang^a^, Qun Yang^a^, Jingxian Ming^a^, Jiawei Zhang^a^, Xiaoxing Yin^a*^, Qian Lu^a*^

^#^These authors contributed equally to this project.

^a^Jiangsu Key Laboratory of New Drug Research and Clinical Pharmacy, Xuzhou Medical University.

^*^Corresponding author: +86-516-83262630; fax: +86-516-83262630

E-mail address: [luqian@xzhmu.edu.cn](mailto:luqian@xzhmu.edu.cn) (Qian Lu), [yinxx@xzhmu.edu.cn](mailto:yinxx@xzhmu.edu.cn) (Xiaoxing Yin)

This document includes:

Figure S1-5

Table S1-3

**Supplemental Figures**


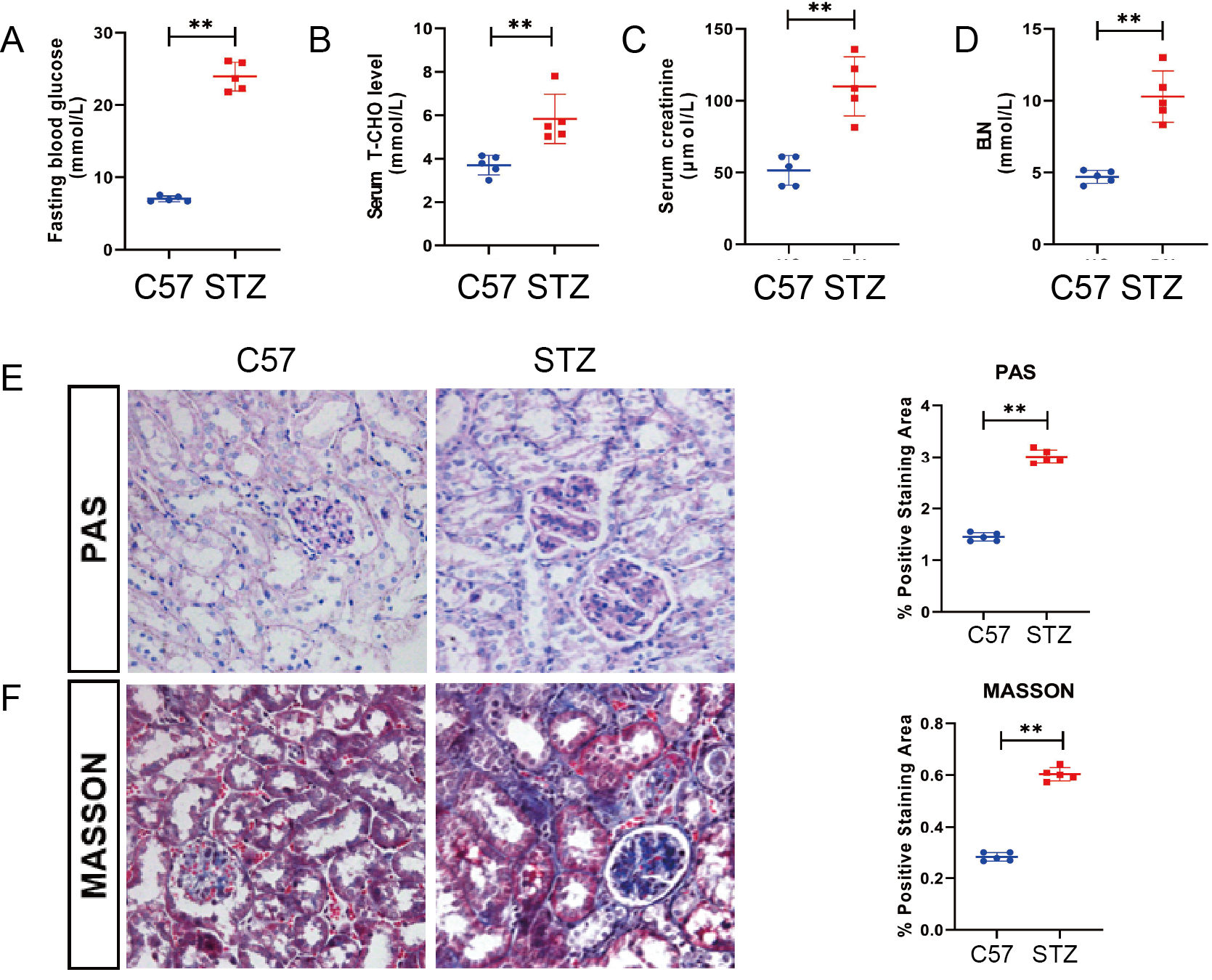


**Figure S1. Establishment and validation of type 2 diabetes mouse model mice.** (A) Fasting blood glucose (FBG) of STZ after injection 72h. n=5. (B-D) The expressions of T-CHO, Cr, and BUN by ELISA. n=5. (E, F) PAS staining (Positive area: red), Masson staining (Positive area: blue) and their quantitative analysis of kidney tissue in mice. Scale bar: 20μm. C57: normal control mice. STZ: STZ diabetic model mice. Data are expressed as mean ± s.e.m, n = 5. Statistical significance was assessed using an unpaired t-test, *^*^P* < 0.05, *^**^P* < 0.01, compared to C57.


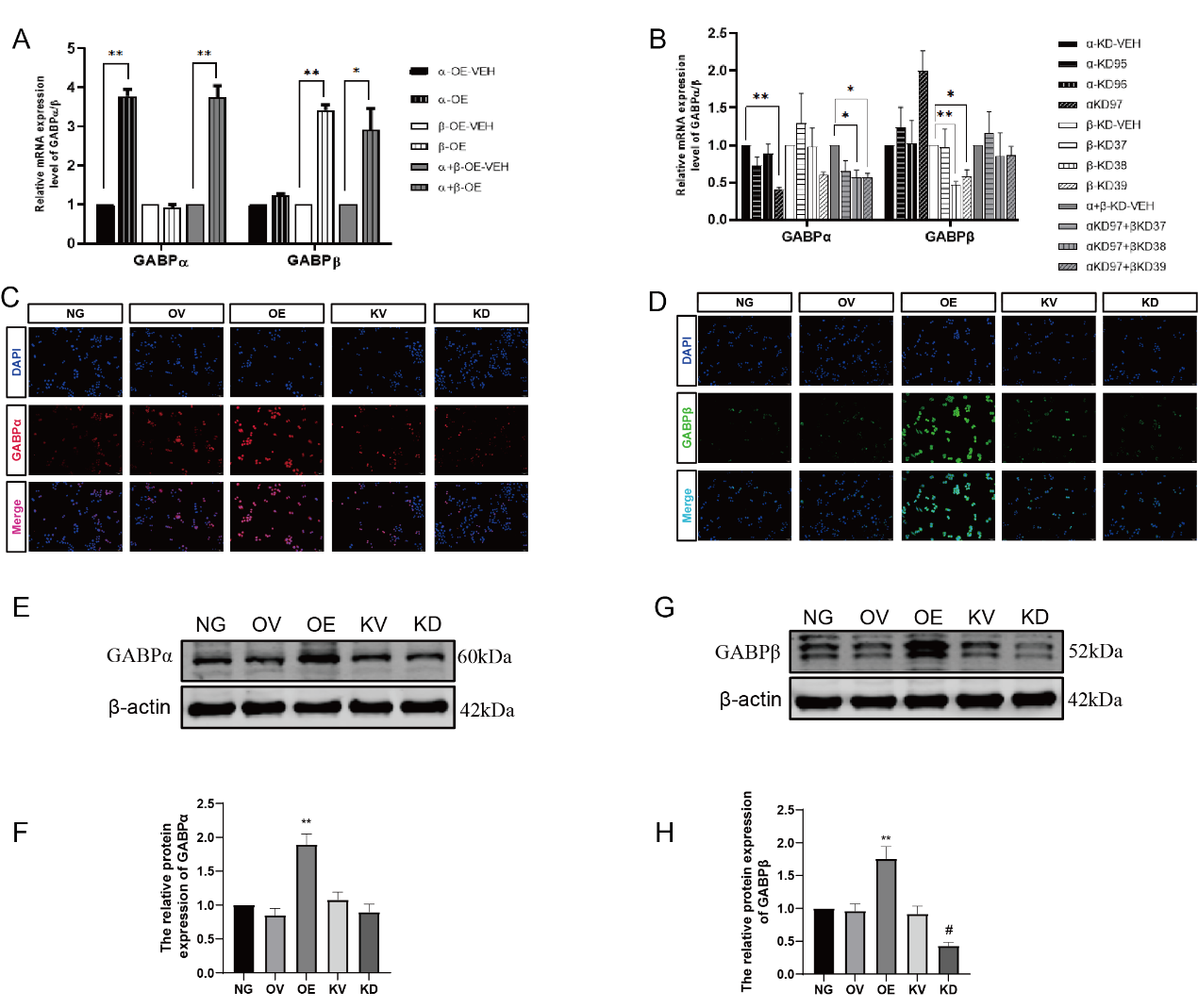


**Figure S2. Establishment of mesangial cells with GABP knockdown and overexpression.** (A, B) The mRNA expression levels of GABPα and GABPβ in mesangial cells. (C, D) Immunofluorescence of GABPα and GABPβ in the mesangial cells (GABPβ, green fluorescence; GABPα ,red fluorescence; Scale bar: 100μm); n = 3. (E, F) The protein expression level of GABPα and GABPβ in mesangial cells by western blot, n = 3. NG: normal mesangial cell; OV: normal mesangial cell with vector; OE: normal mesangial cell with GABPα/b-overexpression lentivirus; KV: normal mesangial cell with vector; KD: mesangial. cell with GABPβ knockdown lentivirs. Data are expressed as mean ± s.e.m, n = 3. Statistical significance was assessed using one-way ANOVA with Tukey’s test, *^*^P* < 0.05, *^**^P* < 0.01, compared to OV; ^#^*P* < 0.05, ^##^*P* < 0.01, compared to KV.


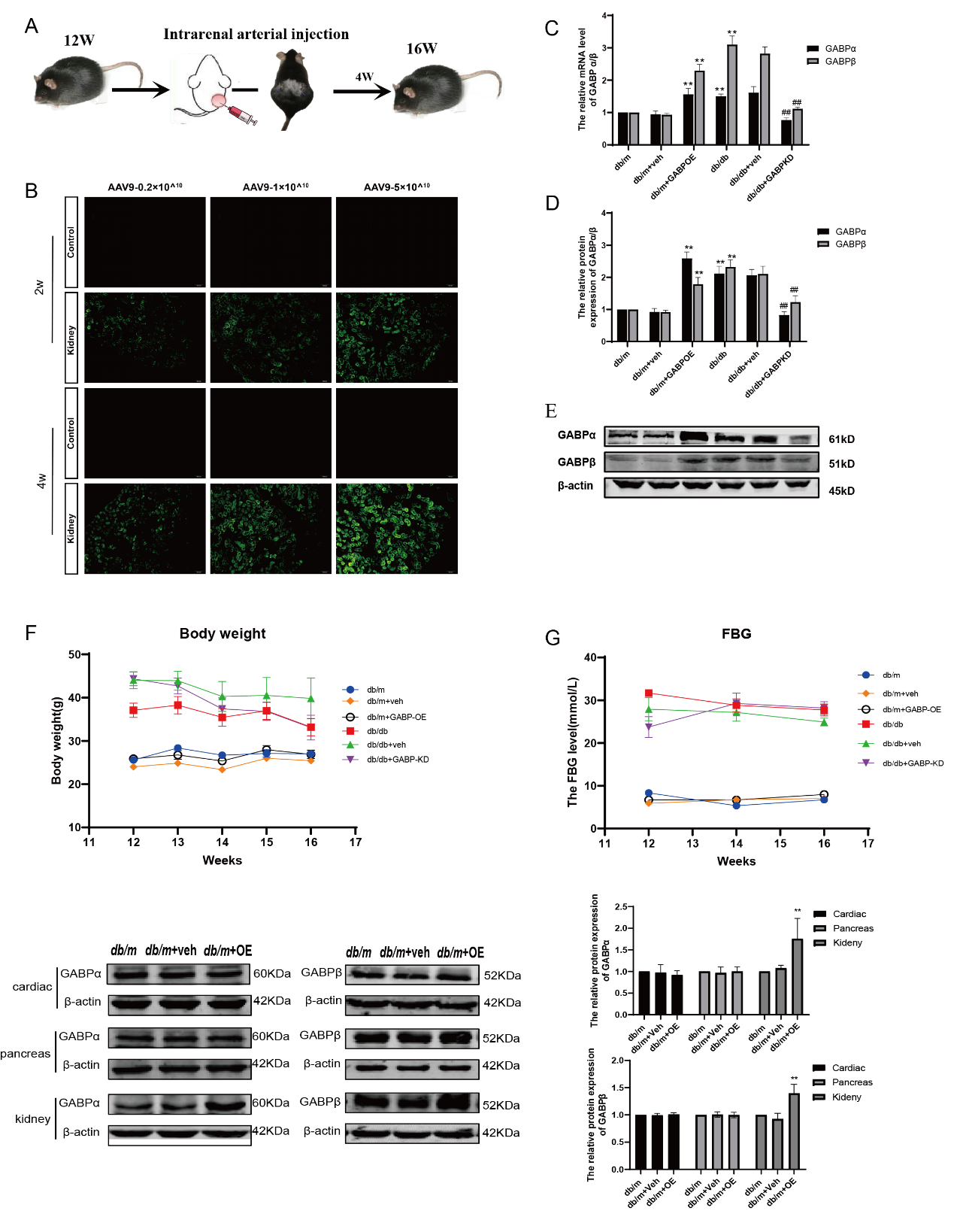


**Figure S3. Establishment of GABP knockdown and overexpression mouse model.** (A) Experimental procedure of GABP Adeno-associated Virus through renal parenchyma injection in mice; (B) Frozen section of mice kidney after renal parenchymal injection; (C, D, E) The protein and mRNA expression levels of GABPα and GABPβ in mesangial cells by western blot, n = 3. (F) Determination of body weight and fasting blood glucose in mice after renal parenchyma injection. (G) The protein expression levels of GABPα and GABPβ expression in cardiac, pancreas and kidney of mice, n = 6. *db/m*: normal control mice; *db/m*+Veh: db/m mice with intra-renal injection of vector; *db/m*+OE: *db/m* mice with intra-renal injection of GABPα/β overexpression adeno-associated virus; db/db: diabetic model mice; db/db+Veh: db/db mice with intra-renal injection of vector, db/db+KD: db/db mice with·intra-renal injection of GABPβ-knockdown adeno-associated·virus. Data are expressed as mean ± s.e.m. Statistical significance was assessed using a one-way ANOVA with Tukey’s test, *^*^P* < 0.05, *^**^P* < 0.01, compared to the *db/m*+Veh, ^#^*P* < 0.05, ^##^*P* < 0.01, compared to the *db/db*+Veh.


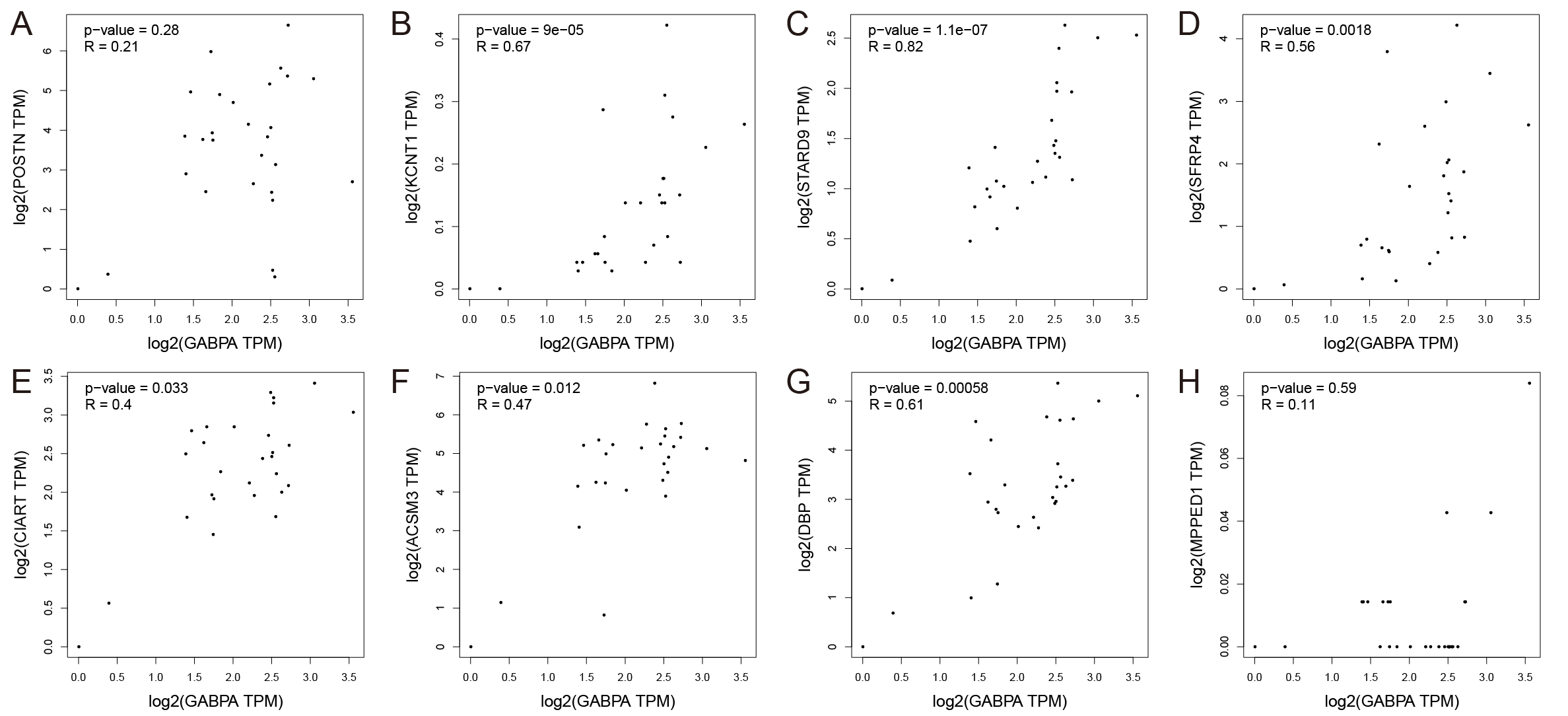


**Figure S4. The correlation analysis between differentially expressed genes and GABPα**. (A-H) The correlation coefficients of GABPα and 8 differentially expressed genes by GEPIA.


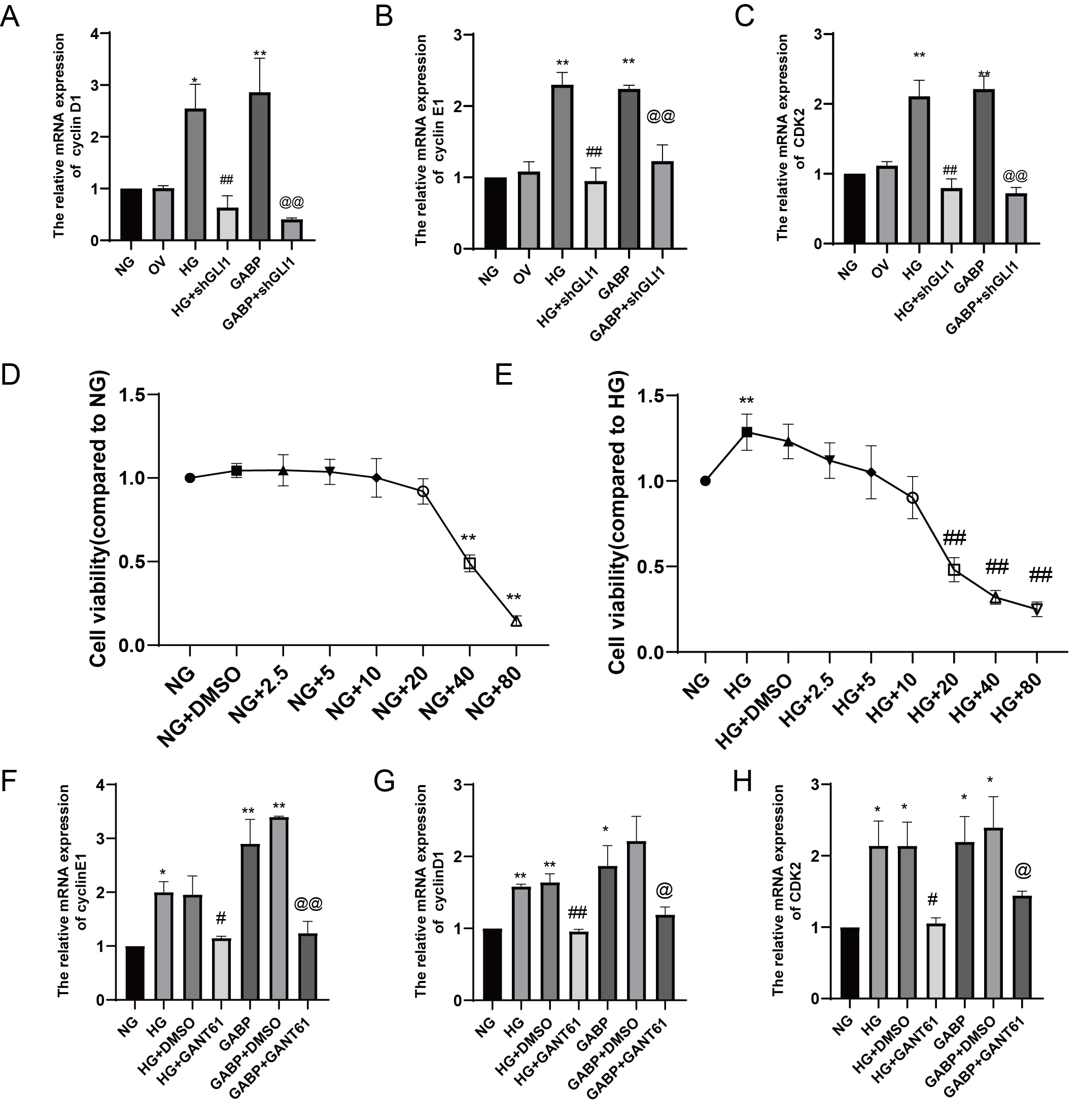


**Figure S5. Inhibition of GLI1 expression prevents GABP-induced mesangial cell proliferation.** (A-C) The mRNA expression levels of Cyclin D1, cyclin E and CDK 2 in mesangial cells. (D, E) The effect of GANT61 on cell proliferation by CCK8. (F-H) The mRNA expression levels of Cyclin D1, cyclin E and CDK 2 in mesangial cells. NG: normal mesangial cell group (5.56 mM glucose concentration), HG: mesangial cell with 30mM glucose group, HG+DMSO: high glucose cultured mesangial cell with DMSO control group, HG+GANT61: high glucose cultured mesangial cell with 10 μM GANT61 group, GABP: normal mesangial cell with GABPα/β-overexpression lentivirus, GABP+DMSO: normal mesangial cell with GABPα/β-overexpression lentivirus and DMSO group, GABP+GANT61: normal mesangial cell with GABPα/β-overexpression lentivirus and 10 μM GANT61 group. Data are expressed as mean ± s.e.m, n = 3. Statistical differences were determined by student t test and one-way ANOVA, *^*^P* < 0.05, ^*^*^*^P* < 0.01, compared to the NG, ^#^*P* < 0.05, ^##^*P* < 0.01, compared to the HG, ^@^*P* < 0.05,^@@^*P* < 0.01, compared to the GABP.

**Supplemental Tables**

**Table S1. Differentially expressed genes with fold changes of GABP overexpression and knockdown in mouse kidney.**

| Gene symbol | *db/m*-veh vs *db/m*-GABP-OE | |  | *db/db*-veh vs *db/db*-GABP-KD | |
| --- | --- | --- | --- | --- | --- |
|  | log_2_FoldChange | *P-*value |  | Down-log_2_FoldChange | *P-*value |
| Sfrp4 | 1.890074566 | 0.001197237 |  | -4.412768775 | 0.039488957 |
| Mpped1 | 1.160455052 | 0.020186818 |  | -5.072263295 | 2.9664E-08 |
| Gli1 | 2.531156226 | 0.015773292 |  | -3.687240642 | 0.009559673 |
| Kcnt1 | 1.45957853 | 0.184255662 |  | -3.596265165 | 2.04355E-08 |
| Acsm3 | 1.26950219 | 0.014043226 |  | -2.716200559 | 0.002927935 |
| Ciart | 1.746727235 | 0.017820593 |  | -2.21936131 | 0.034894424 |
| Postn | 1.297613851 | 0.021255895 |  | -2.417561872 | 0.000432692 |
| Dbp | 1.705756114 | 0.045323931 |  | -1.377337293 | 0.016410736 |
| Stard9 | 1.036571828 | 0.013124813 |  | -1.533878138 | 0.005327473 |

**Table S2. Basic clinical data and serum GABP levels in NC group, DM group, DN group and MGN group**.

| Groups | HC (n=12) | DM (n=13) | DN (n=39) | MGN (n=6) |
| --- | --- | --- | --- | --- |
| Age (years) | 51.5±5.14 | 51.85±8.48 | 58.67±12.53 | 47.17±11.2 |
| Gender (Male/Female) | 7/5 | 10/3 | 27/12 | 5/1 |
| Height (cm) | 167±9.01 | 170.15±6.03 | 166.47±9.76 | 172±4.2 |
| Weight (Kg) | 60.75±6.92 | 71.62±13.28 | 72.66±13.67 | 80.83±23.96 |
| BMI | 21.75±1.41 | 24.79±4.83 | 26.05±3.36 | 27.15±7.18 |
| BUN (mmol/L) | 4.06±0.58 | 4.78±1 | 9.67±6.48* | 5.65±1.65 |
| Blood uric acid (μmol/L) | 293.42±90.78 | 256.46±66.72 | 344.32±99.82* | 414±116.35 |
| CREA (μmol/L) | 59.75±11.22 | 53.46±12.25 | 152.79±152.29* | 81.83±26.87 |
| eGFR (mL/(min×1.73m^2^)) | 119.02±15.05 | 113.86±6.62 | 60.34±36.08** | 83.01±17.96 |
| GABP (pg/mL) | 282.06±97.53 | 322.04±49.99 | 416±111.57* | 345.54±22.85 |

Data are expressed as mean  ±  SD, ^*^*P* < 0.05, ^**^*P* < 0.01.

**Table S3. Primer sequences for qPCR.**

| Description | Primer sequence (5’ to 3’) | |
| --- | --- | --- |
| GABPα  (Mus musculus) | Forward: | GTTCAAGGCAAGAGATTTGTGT |
|  | Reverse: | TGTTCACACTCTATGACCAGAC |
| GABPβ  (Mus musculus) | Forward: | ATCTGCTGTTCAGTTTGGAAAC |
|  | Reverse: | AACTATCGTGATGACTTGCTGA |
| GLI1  (Mus musculus) | Forward: | CCCCTCTCTAGCTTCTATCCACCCAG |
|  | Reverse: | TTTCTCGCTGTTGCCACCCG |
| Cyclin D1  (Mus musculus) | Forward: | GAGGCGGATGAGAACAAGCAGAC |
|  | Reverse: | GAGGCGGATGAGAACAAGCAGAC |
| Cyclin E  (Mus musculus) | Forward: | GATGGCATCAAACAGGGCAAAGTG |
|  | Reverse: | TGTCTGGAGGTGGCTGGTGTAC |
| Cdk2  (Mus musculus) | Forward: | TGCCCGCTGTGCTCCTATCTAG |
|  | Reverse: | GGTCCCCAGAGTCCGAAAGATCC |
| Ptch1  (Mus musculus) | Forward: | ATACATCAGCCTGCGCCACT |
|  | Reverse: | ATGACAATGATCCCGGCCGT |
| Snail  (Mus musculus) | Forward: | CTCGCGAGCGGAGTTGACTA |
|  | Reverse: | TATAGTTGGGCTTCCGGCGG |
| Vegfc  (Mus musculus) | Forward: | ATGCCTGGCTCAGCAGGATT |
|  | Reverse: | CCAGCTCCTTGTTGGGTCCA |
| Vegfd  (Mus musculus) | Forward: | AGGAGTTGCTGCAAATCGCG |
|  | Reverse: | TGGAGCGATGGGATGCTGAG |
| Gapdh  (Mus musculus) | Forward: | GGTTGTCTCCTGCGACTTCA |
|  | Reverse: | TGGTCCAGGGTTTCTTACTCO |
